# Patient-specific risk factors for repair failure and poor functional outcome after rotator cuff repair - an umbrella review

**DOI:** 10.1186/s12891-025-08608-w

**Published:** 2026-02-16

**Authors:** Julia Sußiek, Stefan Buchmann, Knut Beitzel, Sebastian Lappen, Rehabilitation Committee of the German, Austrian and Swiss Shoulder and Elbow Society

**Affiliations:** 1 Department of Trauma-, Hand- and Reconstructive Surgery, University Hospital Muenster, Waldeyerstr. 1, 48149 Muenster, Germany; 2 Department of Orthopedic Sports Medicine, Technical University of Munich, Ismaninger Str. 22, 81675 Munich, Germany; 3 Orthopaedisches Fachzentrum (OFZ) Weilheim, Johann-Baur-Straße 5, 82362 Weilheim in Oberbayern, Germany; 4 ATOS Kliniken Köln, Aachener Str. 1021B, 50858 Cologne, Germany; 5 Department of Orthopaedic Sports Medicine, Klinikum rechts der Isar, Technical University Munich, Ismaninger Str. 22, 81675 Munich, Germany

**Keywords:** Rotator cuff tear, Rehabilitation, Risk factors, Prognostic factors, Cuff integrity, Systematic review

## Abstract

**Purpose:**

Understanding patient-specific factors that influence postoperative outcomes and failure rates following rotator cuff repair is crucial for surgeons to tailor individualized treatments. The purpose of this umbrella review was to identify preoperatively measurable factors that influence the risk of retear and functional outcomes following rotator cuff repair (RCR). Additionally, the study aimed to evaluate the quality of evidence from systematic reviews and meta-analyses to provide a comprehensive understanding of these predictive factors.

**Methods:**

A systematic search of the MEDLINE database via PubMed was conducted to identify systematic reviews and meta-analyses reporting preoperatively measurable factors affecting functional outcomes and failure rates following arthroscopic rotator cuff repair. The methodological quality of included reviews was assessed using the AMSTAR checklist. Data synthesis summarized key risk factors, quantified study overlap via Corrected Covered Area (CCA), and examined heterogeneity and publication bias when reported in the reviews.

**Results:**

Twenty-three systematic reviews, including 11 meta-analyses, met the inclusion criteria, yielding a CCA of 0.403, which reflects moderate overlap. The average AMSTAR score was 7.57, indicating moderate methodological quality. However, only a few reviews included analyses of heterogeneity or publication bias, and the evidence presented was often contradictory. Meta-analyses revealed statistically significant associations between higher retear rates and factors such as advanced age, reduced bone mineral density, elevated body mass index, diabetes, shorter acromiohumeral interval, increased critical shoulder angle, involvement of multiple tendons, greater tendon retraction, longer symptom duration, larger tear size, poor tissue quality, and greater distance from the musculotendinous junction to the glenoid. Genetic analyses provided moderate to strong evidence linking healing failure to mutations in the matrix metallopeptidase 3 (MMP3) and Tenascin C (TNC) genes, as well as a single nucleotide polymorphism (SNP) in the Estrogen Related Receptor β (ESRRβ) gene. Improved tendon healing was associated with the upregulation of the growth factor Bone Morphogenetic Protein 5 (BMP5) and increased expression of collagen type III (COL3). High preoperative expectations consistently correlated with better functional outcomes, whereas other psychological factors, such as concerns and fear avoidance, were associated with poorer outcomes.

**Conclusion:**

Evidence synthesized in this review underscores the importance of patient age, expectations, and the extent of the rotator cuff tear in influencing outcomes following rotator cuff repair. These factors should be carefully considered in treatment planning for patients undergoing rotator cuff repair.

**Supplementary Information:**

The online version contains supplementary material available at 10.1186/s12891-025-08608-w.

## Introduction

Rotator cuff disorders are the leading cause of shoulder pain, accounting for an estimated 65–85% of cases [1]. With an aging population, the incidence of these conditions is anticipated to increase further [2]. The optimal treatment for rotator cuff tears remains a subject of debate. Many patients with symptomatic rotator cuff tears achieve satisfactory results with non-surgical management [3]. However, surgical repair is often indicated for younger individuals or those with high functional demands who sustain acute traumatic tears, as these cases carry a higher risk of tear progression and suboptimal outcomes with conservative treatment [4], and for patients with persistent pain and weakness unresponsive to physical therapy and other non-surgical interventions [5, 6]. Advancements in diagnostic techniques and arthroscopic surgical methods have led to a significant increase in the prevalence of RCR [7], as surgical rates have risen by approximately 200% in both Europe and the United States in recent years [8–10].

While surgical RCR can improve function and alleviate pain in cases of rotator cuff tears [3, 11, 12], failure rates remain substantial, ranging from 7 to 69% despite treatment advancements [13–15]. Revision surgeries pose additional challenges, being more technically demanding, time-intensive, and less effective [16, 17]. Consequently, proactive measures should be implemented to minimize the risk of repair failure and improve primary surgical outcomes. This includes the choice of surgical techniques, such as the selection of sutures, anchors, and bone preparation methods, which are all considered crucial for the success of rotator cuff repair [18]. Additionally, the timing of postoperative rehabilitation—whether early or delayed—may also significantly impact the outcomes of RCR, influencing both tendon healing and functional recovery [19]. However, certain patient-specific factors can also be evaluated preoperatively and should play a role in creating a patient-specific treatment plan. Sociodemographic risk factors, including age, comorbidities, and health behaviors such as smoking and physical activity, are discussed as potential risk factors for retear after RCR [20–22]. Similarly, shoulder-specific factors, such as tear characteristics and tendon quality, have been reported to affect the likelihood of healing after tendon repair [23]. However, it is also known that psychological factors influence the recovery process, and genetic influences have also been described as relevant to the chances of a successful therapy [24–28].

Therefore, this umbrella review was conducted to identify preoperatively measurable factors that influence the risk of retear and functional outcomes following rotator cuff repair, as well as to evaluate the quality of evidence presented in systematic reviews and meta-analyses.

## Methods

### Literature search strategy

A systematic literature search was conducted according to the methodology defined by Smith et al. [29] for conducting a systematic review of systematic reviews in order to identify relevant systematic reviews and meta-analyses investigating preoperatively measurable factors influencing functional outcomes and failure rates after arthroscopic rotator cuff repair. The search was performed using the MEDLINE database via PubMed. The search was limited to articles published in English or German up to June 2022. The search terms were defined according to the Cochrane PICO concept [30] (Table 1). The literature search string is presented in Appendix 1.

**Table 1:** Determination of the search terms according to the PICO concept [30]

		Key Words	Synonyms and other terms
P	Population	Rotator Cuff Tear	Rotator Cuff Injury Rotator Cuff Tear
I	Intervention	Repair	Refixation Arthroscopy Open Surgery Operative surgical procedures Surgery Surgical Procedures Operative
C	Comparison	*Not required*	
O	Outcome	Retear	Risk factor Prognostic Predictive Influence Outcome Rotator Cuff Retear

### Inclusion and exclusion criteria

Articles were included if they met the following criteria: (1) Systematic reviews and meta-analyses written in either English or German, (2) published after January 1st, 2012, and (3) describing preoperatively measurable factors influencing failure rates or poor functional outcome of patients after arthroscopic RCR. Reviews were excluded if they (1) did not contain clearly defined risk factors, (2) were not conducted systematically, or (3) compared different therapy options such as surgical techniques or rehabilitation protocols.

### Study selection

Two independent reviewers (S.L., J.S.) independently screened the titles and abstracts of retrieved articles to assess eligibility for inclusion. Full-text articles of potentially eligible studies were then assessed for final inclusion based on the predetermined inclusion and exclusion criteria. Any discrepancies between reviewers were resolved through consensus discussion.

### Quality assessment

The methodological quality of included systematic reviews and meta-analyses was assessed using the AMSTAR (A MeaSurement Tool to Assess systematic Reviews) checklist [31]. Each study was evaluated based on predefined criteria of an 11-item checklist, each scored as either 0 (“no”, “cannot answer”, or “not applicable”) or 1 (“yes”), and an overall quality score was assigned. Scores ranging from 0 to 3 were considered indicative of low quality, scores from 4 to 7 indicated moderate quality, and scores from 8 to 11 signified high quality.

### Data synthesis

Data synthesis involved summarizing the findings from included systematic reviews and meta-analyses including included study characteristics (e.g., authors, publication year), evidence level, sample size, and patient population characteristics. Key preoperatively measurable risk factors associated with poor functional outcomes and failure rates after RCR were identified and synthesized. Any discrepancies or contradictory findings among studies were noted and discussed. To quantify the level of overlap, the Corrected Covered Area (CCA) was calculated by dividing the number of repeated primary studies across reviews by the total number of unique primary studies multiplied by the total number of reviews minus one.

## Results

### Search results

**Fig. 1:**
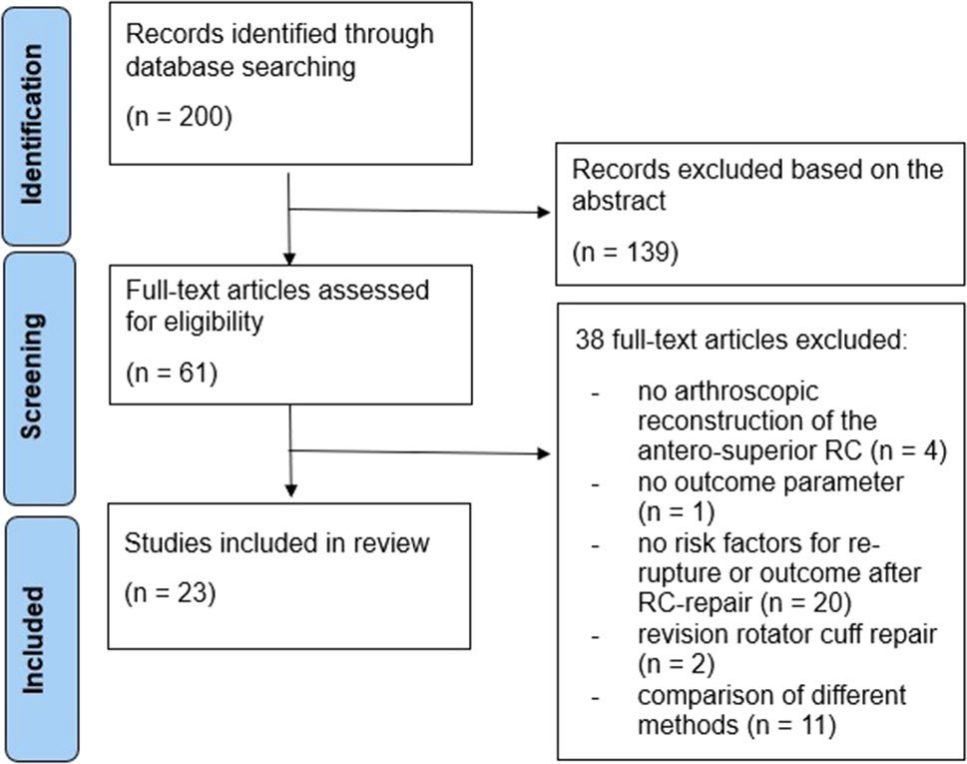
Flowchart of the number of studies identified and included

Initially, a total of 200 records were retrieved and screened against the eligibility criteria. Following the evaluation of titles and abstracts, 139 studies were excluded as they did not meet the predetermined requirements. Subsequently, the full texts of the remaining 61 documents were assessed. Ultimately, 23 documents [20, 23, 32–53] including 11 meta-analyses [20, 23, 32, 37, 40, 43–46, 49, 53] met the predefined inclusion criteria and were included in the review. The specific retrieval process is illustrated in Fig. 1.

A total of 387 primary studies were included across the 23 included reviews (Appendix 2). Of these, 285 were unique primary studies, leading to a CCA of 0.403.

### Literature quality assessment

The AMSTAR score of the included reviews ranged from 4 to 11 points. Eleven reviews were classified as ‘moderate quality’, while 12 studies were rated as ‘high quality’. The mean AMSTAR score calculated across all included literature was 7.57 points, indicating a moderate level of quality.

### Heterogeneity and publication Bias analysis

Only seven of the included reviews provided a heterogeneity analysis. When available, heterogeneity was most often classified as moderate or high (Table 2).

**Table 2:** Summary of heterogeneity analysis of the included reviews

Author	Heterogeneity
Altintas et al. [32]	I^2^ = 61.4%; 95% CI, 27.5-79.4%).
Cimino et al. [33]	No statistical heterogeneity analysis
Coronado et al. [34]	No statistical heterogeneity analysis
Docter et al. [35]	No statistical heterogeneity analysis
Fan et al. [20]	• UCLA: I^2^ = 9% • VAS: I^2^ = 64%) • Retear rate: I^2^ = 31%
Fermont et al. [36]	No statistical heterogeneity analysis
Haunschild et al. [37]	No statistical heterogeneity analysis based on retear rate or functional outcome
Holtedahl et al. [53]	I^2^ = 92.6% in RCT; I^2^ = 84.7% PCS)
Kennedy et al. [39]	No statistical heterogeneity analysis
Khair et al.[40]	No statistical heterogeneity analysis
Kunze et al. [41]	No statistical heterogeneity analysis
Lambers Heerspink et al. [42]	No statistical heterogeneity analysis
Lapner et al. [43]	No statistical heterogeneity analysis based on retear rate or functional outcome
Longo et al. [44]	• Retear rate within the interval 3 < months ≤ 6: I^2^ = 77% • Retear rate within the interval 6 < months ≤ 12: I^2^ = 78% • Retear rate within the interval 12 < months ≤ 24: I^2^ = 88% • Retear rate > 24 months: I^2^ = 56%
Lu et al. [45]	• ASES: I^2^ = 93% • UCLA: I^2^ = 88% • Constant score: I^2^ = 0%
McElvany et al. [46]	No statistical heterogeneity analysis
Mousley et al. [47]	No statistical heterogeneity analysis
Panattoni et al. [48]	No statistical heterogeneity analysis
Saccomanno et al. [49]	No statistical heterogeneity analysis
Santiago-Torres et al. [50]	No statistical heterogeneity analysis
Sheean et al. [51]	I^2^ = 7%
Spennacchio et al. [52]	No statistical heterogeneity analysis
Zhao et al. [23]	• Age: I^2^ = 93% • Male sex: I^2^ = 70 • Female sex: I^2^ = 55 • Dominant arm: I^2^ = 0% • Right shoulders: I^2^ = 0% • Left shoulders: I^2^ = 0% • Level of sports activity: I^2^ = 56% • Body mass index: I^2^ = 62% • Hypertension: I^2^ = 74% • Hyperlipidemia: I^2^ = 0% • Cardiovascular disease: I^2^ = 0% • Diabetes: I^2^ = 0% • Smoking: I^2^ = 35% • Demanding work: I^2^ = 79% • Trauma history: I^2^ = 42% • Subscapularis fatty infiltration, grade < 2: I^2^ = 0% • Subscapularis fatty infiltration, grade > 2: I^2^ = 78% • Supraspinatus fatty infiltration, grade < 2: I^2^ = 87% • Supraspinatus fatty infiltration, grade > 2: I^2^ = 90% • Infraspinatus fatty infiltration, grade < 2: I^2^ = 89% • Infraspinatus fatty infiltration, grade > 2: I^2^ = 80% • Forward flexion: I^2^ = 0% • External rotation: I^2^ = 82% • Internal rotation: I^2^ = 45% • Preoperative Frozen shoulder: I^2^ = 0% • Symptom duration: I^2^ = 28% • Symptom aggravation: I^2^ = 0% • Preoperative VAS: I^2^ = 0% • Preoperative UCLA: I^2^ = 84% • Steroid injection: I^2^ = 86% • Bone mineral density: I^2^ = 84% • Tear length: I^2^ = 92% • Tear width: I^2^ = 91% • Tear size area: I^2^ = 82% • Amount of retraction: I^2^ = 0% • Critical shoulder angle: I^2^ = 0% • Glenoid inclination: I^2^ = 0% • Acromiohumeral interval: I^2^ = 40% • Distance of musculotendinous junction to glenoid: I^2^ = 0%

A statistical assessment of publication bias was conducted in only three of the included reviews, with significant evidence of publication bias observed solely in randomized controlled trials [53] and for variables related to male and female sex [23] (Table 3).

**Table 3:** Summary of publication Bias analysis of the included reviews

Author	Heterogeneity
Altintas et al. [32]	Funnel plot analysis and the rank correlation test did not reveal significant evidence for publication bias (tau = 0.242, *p* = .311)
Cimino et al. [33]	No publication bias assessment
Coronado et al. [34]	No publication bias assessment
Docter et al. [35]	No publication bias assessment
Fan et al. [20]	No publication bias assessment
Fermont et al. [36]	No publication bias assessment
Haunschild et al. [37]	No publication bias assessment
Holtedahl et al. [53]	Begg and Mazumdar’s rank correlation test was significant in the RCT but not in the PCS subgroup (Kendall’s tau = 0.47, *p* < .0001; tau = 0.24, *p* = .14, respectively).
Kennedy et al. [39]	No publication bias assessment
Khair et al.[40]	No publication bias assessment
Kunze et al. [41]	No publication bias assessment
Lambers Heerspink et al. [42]	No publication bias assessment
Lapner et al. [43]	No publication bias assessment
Longo et al. [44]	No publication bias assessment
Lu et al. [45]	No publication bias assessment
McElvany et al. [46]	No publication bias assessment
Mousley et al. [47]	No publication bias assessment
Panattoni et al. [48]	No publication bias assessment
Saccomanno et al. [49]	No publication bias assessment
Santiago-Torres et al. [50]	No publication bias assessment
Sheean et al. [51]	No publication bias assessment
Spennacchio et al. [52]	No publication bias assessment
Zhao et al. [23]	Egger test showed no publication bias for age (*p* = .81), tear length (*p* = .11), symptom duration (*p* = .37), dominant hand (*p* = .07), diabetes (*p* = .89), and smoking (*p* = .56). Male sex (*p* = 03) and female sex (*p* = .03) showed publication bias.

### Sociodemographic risk factors

**Fig. 2:**
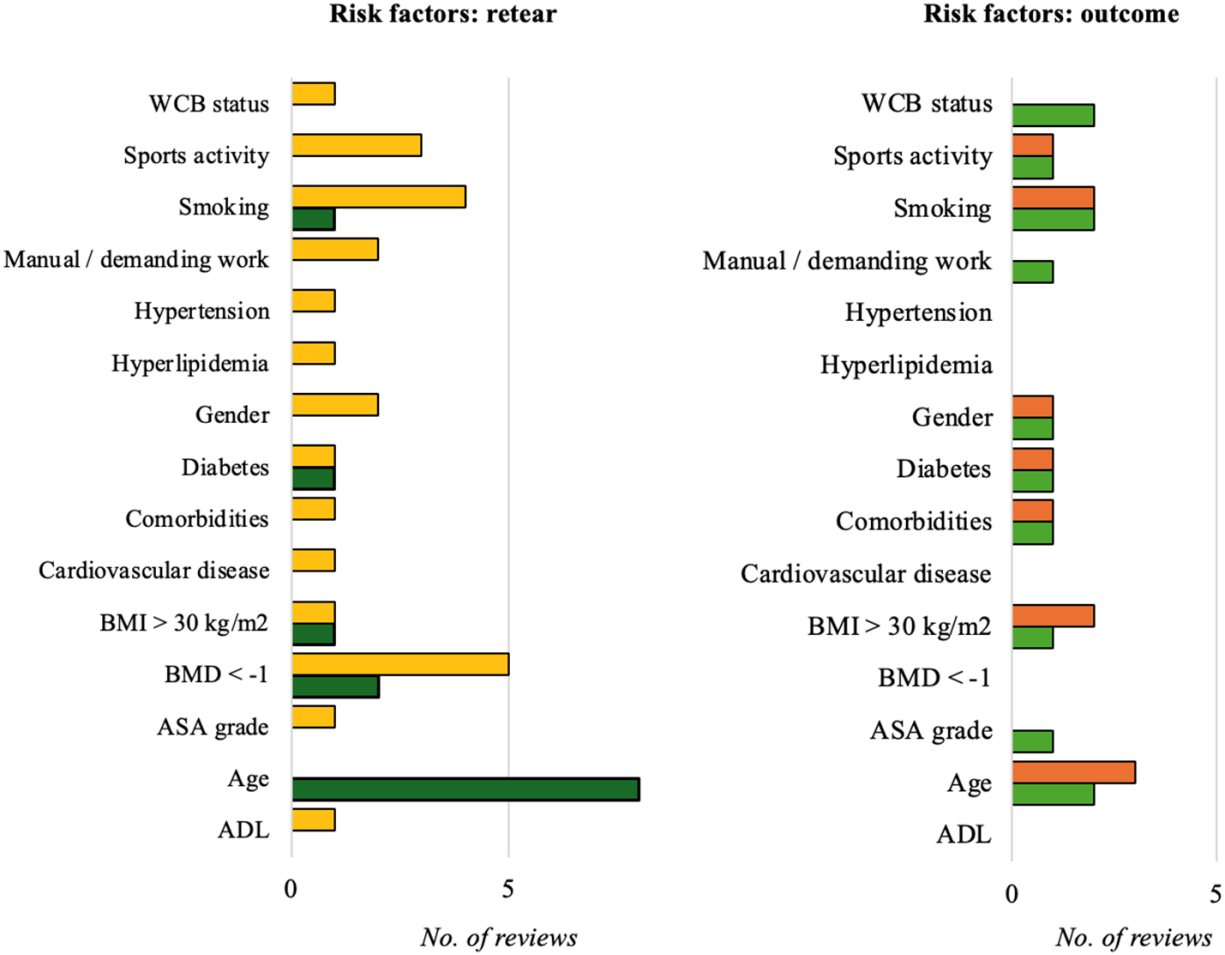
Sociodemographic risk factors for retear (left) and functional outcomes (right) after rotator cuff repair. Age was the factor most frequently associated with retear rates, with the majority of reviews identifying a clear association (dark green). For most other factors, no or mixed evidence was reported (yellow). Only age, smoking, and workers’ compensation status (WCB) were supported by more than one review indicating a clear association with functional outcome (light green). However, for most of these factors, other reviews reported either no evidence or mixed findings (orange)

Fourteen sociodemographic risk factors for retear or poor functional outcomes after RCR were identified in the included reviews, with evidence often being contradictory (Table 4; Fig. 2).

**Table 4:** Sociodemographic risk factors identified in the included reviews

	Association with retear rate (no. of reviews)		Association with outcome (no. of reviews)	
	Evident Association	No or Mixed Evidence	Evident Association	No or Mixed Evidence
ADL		1 [49]		
Age	8 [23, 36, 38, 42, 44, 46, 49, 52]		2 [36, 52]	3 [32, 42, 49]
ASA grade		1 [49]	1 [49]	
BMD < -1	2 [23, 36]	1 [49]		
BMI > 30 kg/m^2^	1 [23]	1 [49]	1 [36]	2 [42, 49]
Cardiovascular disease		1 [23]		
Comorbidities		1 [49]	1 [49]	1 [42]
Diabetes	1 [23, 45]	1 [49]	1 [36, 45]	1 [45, 49]
Gender		2 [23, 49]	1 [36]	1 [49]
Hyperlipidemia		1 [23]		
Hypertension		1 [23]		
Manual / demanding work		2 [23, 49]	1 [49]	
Smoking	1 [20]	4 [23, 42, 49, 50]	2 [20, 50]	2 [42, 49]
Sports activity		3 [23, 32, 49]	1 [36]	1 [32]
WCB status		1 [49]	2 [42, 49]	

ADL, activities of daily living; BMD, bone mineral density; BMI, body mass index; WCB, workers’ compensation board

However, meta-analyses showed a statistically significant effect of age [23, 44, 46, 49, 53], BMD [23], BMI [23], and diabetes [23] on retear rates (Table 5). The results for smoking were inconsistent even among meta-analyses, with one analysis finding that smoking increased retear risk [20], while two other did not observe this effect [23, 49]. Additionally, smoking was found to negatively impact the postoperative Constant score, but no significant effect was observed on the American Shoulder and Elbow Surgeons (ASES), Simple Shoulder Test (SST), University of California, Los Angeles (UCLA), or Visual Analog Scale (VAS) scores [20].

**Table 5:** Impact of sociodemographic factors on retear rates and functional outcomes, as reported in the included meta-analyses

Category	Study	Results
**Statistically significant**		
Age	Holtedahl et al. [53]	Higher age is a risk factor for retear • Univariant analysis: regression coefficient = 0.076 (SE = 0.027; *p* = .007; R² = 0.18) • Multivariate analysis: regression coefficient = 0.067 (SE = 0.027; *p* = .020; R² = 0.22)
	Longo et al. [44]	Older age is associated with higher retear rate • OR = 1.8 (95% CI: 1.5, 2.3; *p* < .0001)
	McElvany et al. [46]	Older age is associated with higher retear rates • OR = 1.67 (95% CI: 1.49, 1.87; *p* < .001)
	Saccomanno et al. [49]	Retear risk was found to be significantly increased by older age • OR = 1.10 (95% CI: 1.05, 1.15; *p* < .05)
	Zhao et al. [23]	Advanced age is a risk factor for rotator cuff retear • WMD = 4.38 (95% CI: 2.16, 6.61; *p* < .001)
BMD	Zhao et al. [23]	Bone mineral density is a risk factors for rotator cuff retear • WMD = -0.56 (95% CI: -1.04, -0.08; *p* = .020)
BMI	Zhao et al. [23]	The higher the BMI, the more likely the rotator cuff retear • WMD = 0.52 (95% CI: 0.23, 0.82; *p* < .001)
Diabetes	Lu et al. [45]	The postoperative Constant-Murley scores of the Non-diabetes group were significantly higher than the diabetes group • WMD = -3.46 (95% CI: -5.33, -1.59; *p* < .001)
	Zhao et al. [23]	Diabetes is a risk factor for postoperative rotator cuff retear • OR = 1.42 (95% CI: 1.02, 1.97; *p* = .040)
Smoking	Fan et al. [20]	Smoking was significantly associated with an increased risk of retear • Risk ratio = 2.06 (95% CI: 1.30, 3.28, I^2^ = 31%; *p* = .002) Smoking was significantly associated with a lower postoperative Constant score • (I^2^ = 0%, *p* = .005)
**Not statistically significant**		
Age	Altintas et al. [32]	Age does not influence the return to sport rate: • β = 0.007; 95% CI, 20.025 to 0.039; *p* = .685
	Holtedahl et al. [53]	No association between clinical outcome and mean age • Univariant analysis: regression coefficient = 0.015 (SE = 0.040; *p* = .69; R² = − 0.01)
Cardivascular disease	Zhao et al. [23]	Cardiovascular disease does not cause rotator cuff retear after rotator cuff repair • OR = 1.33 (95% CI: 0.77, 2.31; *p* = .300)
Diabetes	Lu et al. [45]	No significant difference in postoperative ASES and UCLA scores between diabetics and non-diabetics • ASES: WMD: -9.17 (95% CI, -22.50, 4.16; *p* = .18) • UCLA: WMD = -1.09 (95% CI: -3.33, 1.16; *p* = .34)
Gender	Zhao et al. [23]	Neither female nor male sex causes rotator cuff retear after rotator cuff repair • Female: OR = 0.85 (95% CI: 0.71, 1.23; *p* = .080) • Male: OR = 0.83 (95% CI: 0.58, 1.20; *p* = .330)
Hyperlipidemia	Zhao et al. [23]	Hyperlipidemia does not cause rotator cuff retear after rotator cuff repair • OR = 1.50 (95% CI: 0.99, 2.26; *p* = .050)
Manual / demanding work	Zhao et al. [23]	Demanding work is not associated with rotator cuff retear • OR = 1.50 (95% CI: 0.64, 3.51; *p* = .350)
Smoking	Fan et al. [20]	No significant differences between smokers and nonsmokers in following scores: • ASES score (I^2^ = 0%, *p* = .100) • SST score (I^2^ = 20%, *p* = .190) • UCLA score (I^2^ = 9%, *p* = .090) • VAS score (I^2^ = 64%, *p* = .190)
Smoking	Zhao et al. [23]	Smoking does not cause rotator cuff retear after rotator cuff repair • OR = 1.02 (95% CI: 0.75, 1.39; *p* = .910)
Sports activity	Zhao et al. [23]	Level of sports activity is not associated with rotator cuff retear • OR = 0.85 (95% CI: 0.42, 1.69; *p* = .640)

SE, standard error; CI, confidence interval; OR, odds ratio; WMD, weighted mean difference; BMD, bone mineral density; BMI, body mass index; ASES, American Shoulder and Elbow Surgeons; SST, Simple Shoulder Test; UCLA, University of California, Los Angeles; VAS, Visual Analog Scale

### Genetic risk factors

One source [47] reviewed over 100 genetic factors potentially influencing healing after RCR. Table 6 outlines factors for which moderate to strong evidence was found. Strong evidence was defined as findings supported by more than two studies with high-quality assessment scores and generally consistent results. Moderate evidence was characterized by either one high-quality study alongside multiple moderate-quality studies or by more than two low-quality studies, provided their findings were generally consistent.

Strong evidence supported an association between failure to heal and mutations in the matrix metallopeptidase 3 (MMP3) and Tenascin C (TNC) genes, as well as one single nucleotide polymorphism (SNP) of the Estrogen Related Receptor β (ESRRβ). Additionally, improved healing was associated with upregulation of the growth factor Bone Morphogenetic Protein 5 (BMP5) and increased expression of collagen type III (COL3), with strong and moderate evidence, respectively.

**Table 6:** Genetic factors showing strong to moderate evidence in influencing healing after RCR

	Failed Healing	Improved Healing
Increased MMP-3 expression	Strong evidence [47]	
Increased COL3 expression		Moderate evidence [47]
Increased TNC expression	Strong evidence [47]	
One SNP of ESRRβ	Strong evidence [47]	
Upregulation of BMP5		Strong evidence [47]

MMP3, Matrix Metallopeptidase 3; TNC: Tenascin C; COL3, Collagen Type III; ESRRβ, Estrogen Related Receptor β; BMP, Bone Morphogenic Protein 5

### Psychological risk factors

Four sources provided information on the influence of psychological factors on functional outcome after RCR [34, 39, 48, 49]; however, none were meta-analyses. High preoperative expectations were identified as the only factor consistently associated with improved functional outcomes across all reviews [34, 39, 48, 49]. All factors reported as relevant are summarized in Table 7.

**Table 7:** Psychological factors influencing functional outcome after RCR

	Influence on functional outcome		
Low Emotional / mental health	Negative [39, 48]		
High MCS of the SF-36	Negative [39]		
Depression	Negative [48]	No effect [39]	
Concerns	Negative [34, 48]		
Stress	Negative [48]		
Pain catastrophizing	Negative [48]		
Kinesiophobia	Negative [48]		
Anxiety	Negative [48]	No effect [39]	
Fear avoidance	Negative [34, 48]		
High preoperative expectations			Positive [34, 39, 48, 49]

MCS, Mental Component Subscale; SF-36, Short Form 36

### Shoulder-specific risk factors

**Fig. 3:**
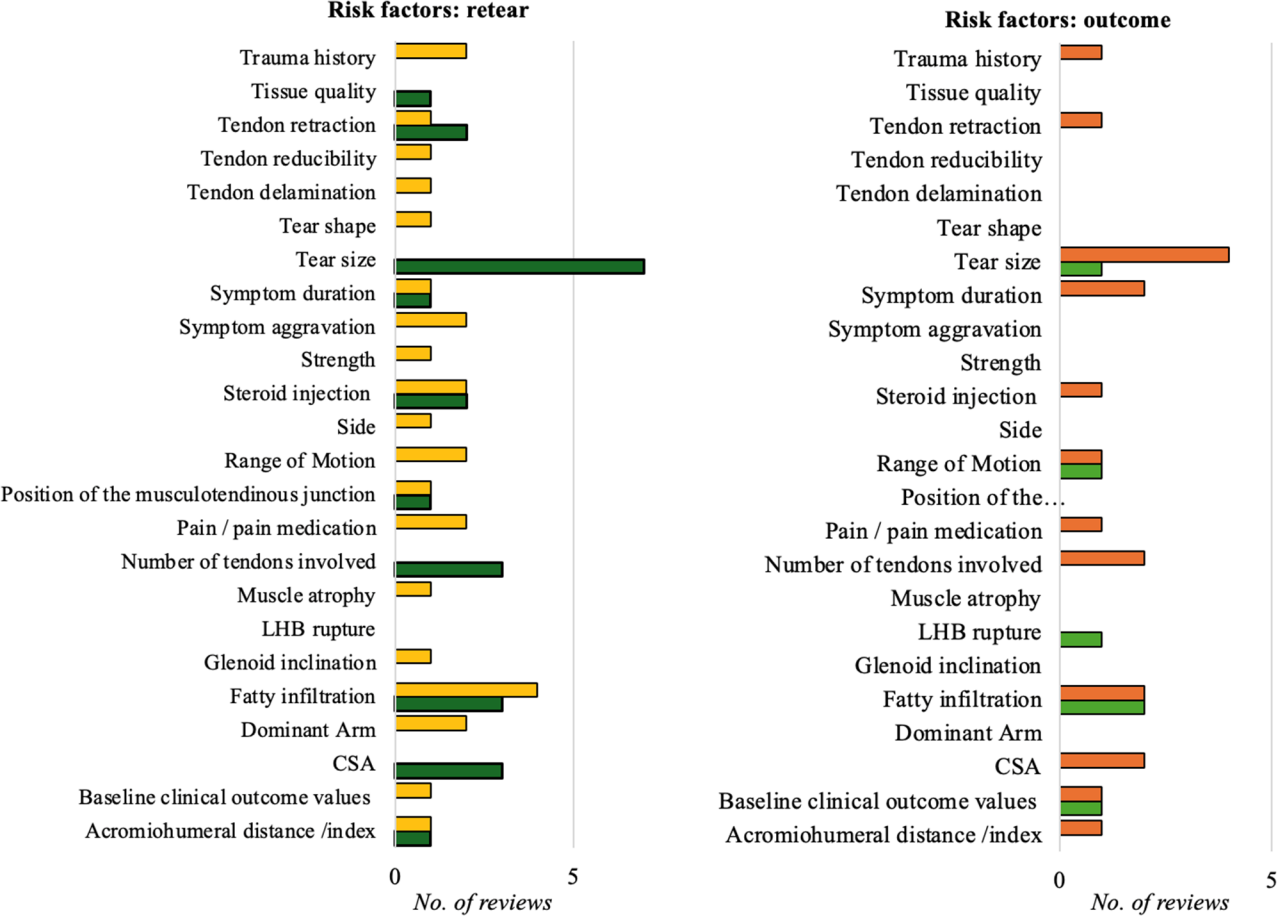
Shoulder-specific risk factors for retear (left) and functional outcomes (right) after rotator cuff repair. The factor most frequently associated with retear rates was tear size, with the majority of reviews identifying a clear association (dark green). For most other factors, no or mixed evidence was reported (yellow). An evident association with functional outcome (light green) was reported for factors such as tear size, range of motion or fatty infiltration. However, for most of these factors, other reviews reported either no evidence or mixed findings (orange)

The included reviews identified twenty-four shoulder-specific risk factors associated with retear or poor functional outcomes following RCR, though the supporting evidence was frequently inconsistent (Table 8; Fig. 3).

**Table 8:** Shoulder-specific factors influencing retear rate and functional outcome after RCR

	Association with retear rate (no. of reviews)		Association with outcome (no. of reviews)	
	Evident Association	No or Mixed Evidence	Evident Association	No or Mixed Evidence
Acromiohumeral distance /index	1 [23]	1 [49]		1 [49]
Baseline clinical outcome values		1 [23, 49]	1 [53]	1 [49]
CSA	3 [23, 35, 51]			2 [35, 51]
Dominant Arm		2 [23, 49]		
Fatty infiltration	3 [36, 40, 46]	4 [23, 42, 44, 49]	2 [46, 49]	2 [40, 42]
Glenoid inclination		1 [23]		
LHB rupture			1 [49]	
Muscle atrophy		1 [49]		
Number of tendons involved	3 [36, 42, 49]			2 [42, 49]
Pain / pain medication		2 [23, 49]		1 [49]
Position of the musculotendinous junction	1 [23]	1 [49]		
Range of Motion		2 [23, 49]	1 [36]	1 [49]
Side		1 [23]		
Steroid injection	2 [33, 41]	2 [23, 49]		1 [33]
Strength		1 [49]		
Symptom aggravation		2 [23, 49]		
Symptom duration	1 [23]	1 [42]		2 [42, 49]
Tear size	7 [23, 36, 38, 42, 44, 46, 49]		1 [36]	4 [38, 42, 49, 52]
Tear shape		1 [49]		
Tendon delamination		1 [49]		
Tendon reducibility		1 [49]		
Tendon retraction	2 [23, 36]	1 [49]		1 [49]
Tissue quality	1 [49]			
Trauma history		2 [42, 49]		1 [42]

CSA, Critical shoulder angle; LHB, Long head of the biceps

Several factors were revealed in meta-analyses to significantly affect retear rates, including the acromiohumeral interval [23], critical shoulder angle [23], involvement of multiple tendons [49], distance from the musculotendinous junction to the glenoid [49], longer symptom duration [49], larger tear size [23, 44, 46, 49, 53], tendon retraction [23], and poor tissue quality [49] (Table 9). Evidence on the role of fatty infiltration was mixed: while two meta-analyses found no link between fatty infiltration and retear rates [44] or tendon healing [49], one study associated higher global fatty infiltration indices—particularly in the supraspinatus and infraspinatus—with increased retear rates [46]. Another meta-analysis highlighted fatty infiltration of the subscapularis or infraspinatus as risk factors, but not of the supraspinatus [23]. Additionally, poor baseline clinical outcome scores were linked to greater improvements in postoperative outcomes [53], although they did not correlate with retear rates [23, 49].

**Table 9:** Impact of shoulder-specific factors on retear rates and functional outcomes, as reported in the included meta-analyses

Category	Study	Results
Statistically significant		
Acromiohumeral distance / index	Zhao et al. [23]	Acromiohumeral interval is a risk factors for rotator cuff retear • WMD = 1.45 (95% CI: 1.59, 1.31; *p* < .001)
Baseline clinical outcome values	Holtedahl et al. [53]	Worse (poorer) outcome value at baseline were associated with better clinical outcomes • Univariant analysis: regression coefficient = − 0.074 (SE = 0.014; *p* < .0001; R² = 0.32) • Multivariate analysis: regression coefficient = 0.088 (SE = 0.013; *p* < .0001; R² = 0.46)
CSA	Zhao et al. [23]	CSA is a risk factors for rotator cuff retear • WMD = 1.66 (95% CI: 0.76, 2.57; *p* = .003)
Fatty infiltration	McElvany et al. [46]	More fatty infiltration was associated with higher retear rates • Supraspinatus, per 1 grade: OR = 2.84 (95% CI: 2.09, 3.85; *p* < .001) • Infraspinatus, per 1 grade: OR = 2.48 (95% CI: 1.11, 5.55; *p* < .001) • Global index, per 1 grade: OR = 1.72 (95% CI: 1.43, 2.06; *p* < .001)
	Zhao et al. [23]	Risk factors for retear were subscapularis and infraspinatus fatty infiltration • Subscapularis fatty infiltration, grade < 2: OR = 0.23 (95% CI: 0.16, 0.33; *p* < .001) • Subscapularis fatty infiltration, grade ≥ 2: OR = 3.37 (95% CI: 1.43, 7.93; *p* = .005) • Infraspinatus fatty infiltration, grade < 2: OR = 0.08 (95% CI: 0.02, 0.29; *p* < .001) • Infraspinatus fatty infiltration, grade ≥ 2: OR: 11.02 (95% CI: 4.30, 28.24; *p* < .001)
Number of tendons involved	Saccomanno et al. [49]	Retear risk was found to be significantly increased by multiple tendons involvement • OR = 7.79 (95% CI: 3.48, 17.47; *p* > .05)
Position of the musculotendinous junction	Zhao et al. [23]	The distance of the musculotendinous junction to the glenoid is a risk factors for rotator cuff retear • WMD = -4.67 (95% CI: -5.05, -4.29; *p* < .001)
Symptom duration	Zhao et al. [23]	The longer the duration of symptoms, the more likely rotator cuff retear • WMD = 4.09 (95% CI: 2.34, 5.85; *p* < .001)
Tear size	Holtedahl et al. [53]	Larger tear size and higher proportion of large-massive tears were associated with increased retear rates • Mean tear size, univariant analysis: regression coefficient = 0.038 (SE = 0.015; *p* = .019; R² = 0.16) • Proportion of small tears, univariant analysis: regression coefficient = -2.425 (SE = 0.765; *p* = .004; R² = 0.19) • Proportion of small tears, multivariant analysis: regression coefficient = -2.840 (SE = 0.754; *p* = .0009; R² = 0.36) Better clinical outcome was associated with a lower proportion of large-massive tear • Proportion of small-medium tears, univariant analysis: regression coefficient = 2.605 (SE = 0.698; *p* = .0008; R² = 0.31) • Proportion of small-medium tears, multivariant analysis: regression coefficient = 2.392 (SE = 0.819; *p* = .007; R² = 0.08)
	Longo et al. [44]	Tear size (small-to-medium tears vs. large-to-massive tears) is associated with higher retear rate • OR = 0.3 (95% CI: 0.2, 0.5; *p* < .0001)
	McElvany et al. [46]	Larger tear size was associated with higher retear rates • ‘‘Large’’ (3–5 cm wide, full supraspinatus and partial infraspinatus tears, or at the glenoid rim) and ‘‘massive’’ (> 5 cm wide, complete supraspinatus and infraspinatus tears, and medial to the glenoid rim) tears vs. “small” (< 1 cm in anteroposterior width, ‘partial-width supraspinatus tear, or tendon edge lateral to the apex of the humeral head) and “medium” (1–3 cm wide, full-width supraspinatus tear, or at the apex of the humeral head) tears: OR = 4.06 (95% CI: 3.08, 5.36; p < .001) • Tear size, per 1 category: OR = 2.62 (95% CI: 2.13, 3.22; *p* < .001)
	Saccomanno et al. [49]	Retear risk was found to be significantly increased by larger tear size • OR = 1.88 (95% CI: 1.32, 2.67; *p* > .05)
	Zhao et al. [23]	Tear length, tear width and tear size are are all risk factors of rotator cuff retear • Tear length: WDM = 0.83 (95% CI: 0.48, 1.18; *p* < .001) • Tear width: WDM = 0.62 (95% CI: 0.26, 0.98 *p* = .007) • Tear size area: WDM = 3.58 (95% CI: 2.31, 5.40; *p* < .001)
Tendon retraction	Zhao et al. [23]	Tendon retraction is a risk factors of rotator cuff retear • WDM = 1.13 (95% CI: 1.07, 1.19; *p* < .001)
Tissue quality	Saccomanno et al. [49]	Retear risk was found to be significantly increased by poor tissue quality • OR = 2.63 (95% CI: 1.06, 6.51; *p* > .05)
**Not statistically significant**		
Baseline score values	Saccomanno et al. [49]	Baseline ASES scores were not associated with retear rates • OR = 1.01 (95% CI: 0.97, 1.05; *p* > .05)
	Zhao et al. [23]	Preoperative VAS scores were not associated with retear rates • WMD = 0.30 (95% CI: -0.03, 0.63; *p* = .070) Preoperative UCLA scores were not associated with retear rates • WMD = 0.35 (95% CI: -2.27, 2.96; *p* = .800)
Dominant arm	Zhao et al. [23]	The dominant arm does not cause rotator cuff retear after rotator cuff repair • OR = 0.89 (95% CI: 0.68, 1.16; *p* = .380)
Fatty infiltration	Longo et al. [44]	Fatty infiltration is not associated with higher retear rate • OR = 0.9 (95% CI: 0.4, 1.9; *p* = .7588)
	Saccomanno et al. [49]	Fatty infiltration did not show a significant correlation with tendon healing • OR = 6.93 (95% CI: 0.26, 187.28; *p* > .05)
	Zhao et al. [23]	Fatty infiltration of the supraspinatus was no risk factors for rotator cuff retear • Supraspinatus fatty infiltration, grade < 2: OR = 0.50 (95% CI: 0.15, 1.70; *p* = .270) • Supraspinatus fatty infiltration, grade ≥ 2: OR = 1.88 (95% CI: 0.51, 6.96; *p* = .350)
Glenoid inclination	Zhao et al. [23]	Glenoid inclination was no risk factors for rotator cuff retear • WMD = 2.39 (95% CI: -0.13, 4.92; *p* = .060)
Pain / pain medication	Saccomanno et al. [49]	Pain and NSAIDs did not significantly affect the retear rate • Pain: OR = 1.69 (95% CI: 0.58, 4.92; *p* < .05) • NSAIDs: OR = 0.72 (95% CI: 0.72, 2.69; *p* > .05)
	Zhao et al. [23]	Preoperative VAS scores were not associated with retear rates • WMD = 0.30 (95% CI: -0.03, 0.63; *p* = .070)
Range of motion	Zhao et al. [23]	Forward flexion, external rotation, internal rotation, and preoperative frozen shoulder were not risk factors for rotator cuff retear • Forward flexion: WMD = -3.53 (95% CI: -9.12, 2.07; *p* = .220) • External rotation: WMD = 0.26 (95% CI: -7.08, 7.60; *p* = .940) • Internal rotation: WMD = -0.32 (95% CI: -0.91, 0.26; *p* = .280) • Preoperative frozen shoulder: OR = 0.99 (95% CI: 0.60, 1.62; *p* = .960)
Side	Zhao et al. [23]	The side of the affected shoulder was no risk factors for rotator cuff retear • Right shoulder: OR = 1.03 (95% CI: 0.81, 1.31; *p* = .820) • Left shoulder: OR = 0.97 (95% CI: 0.77, 1.23; *p* = .820)
Steroid injection	Zhao et al. [23]	Steroid injection was no risk factor for rotator cuff retear • OR = 1.60 (95% CI: 0.34, 7.41; *p* = .550)
Symptom aggravation	Zhao et al. [23]	Symptom aggravation was no risk factor for rotator cuff retear • OR = 1.65 (95% CI: -0.96, 4.27; *p* = .220)

WMD, weighted mean difference; CI, confidence interval; SE, standard error; CSA, Critical shoulder angle; OR, odds ratio; ASES, American Shoulder and Elbow Surgeons; VAS, Visual Analog Scale; UCLA, University of California, Los Angeles

## Discussion

Understanding preoperative factors that influence retear risk and functional outcomes after rotator cuff repair is essential for tailoring treatment to individual patients. This umbrella review revealed the following key findings: (1) the overall quality of evidence was moderate, with variability and inconsistencies partly due to a lack of statistical analyses; (2) meta-analyses identified age, reduced bone mineral density (BMD), high body mass index (BMI), and diabetes as significant sociodemographic factors increasing retear risk, while evidence regarding smoking was contradictory; (3) genetic factors involved in extracellular matrix regulation, such as MMP-3, COL3, and TNC, were crucial for tendon repair, with an SNP in ESRRβ linked to healing failure via increased apoptosis and BMP5 upregulation associated with successful repairs; (4) high preoperative expectations positively influenced outcomes, whereas preoperative concerns, stress, pain catastrophizing, kinesiophobia, anxiety, and fear avoidance were linked to poorer recovery; and (5) larger tear size, multiple tendon involvement, tendon retraction, poor tissue quality, longer symptom duration, fatty infiltration, increased critical shoulder angle (CSA), and reduced acromiohumeral interval were associated with higher retear rates but had no clear impact on functional outcomes.

### Evidence analysis

The mean AMSTAR score calculated across all included literature was 7.57 points, indicating a moderate level of quality. However, many reviews lacked a statistical analysis. Heterogeneity assessments was conducted by only few of the included reviews. Where heterogeneity was analyzed, results often indicated moderate to high variability. Zhao et al. [23] reported substantial heterogeneity associated with demographic factors, such as age (I² = 93%) and male sex (I² = 70%), reflecting variability in sociodemographic factors, anatomic conditions, and intraoperative factors among the study populations. Differences in study desiegn also contributed to heterogeneity [53]. This may be attributed to the use of strict protocols, standardized interventions, and controlled follow-up measures in RCTs to minimize bias, whereas PCS often encompass broader populations with diverse treatments and outcomes. Such real-world variability, coupled with less consistent data collection methods in cohort studies, introduces greater inconsistency and contributes to the observed heterogeneity in meta-analyses. Future meta-analyses should therefore incorporate subgroup analyses and meta-regressions to better understand variability across studies. Additionally, multivariate models could help identify independent predictors and support more personalized risk assessment. These methods would enhance the robustness and clinical applicability of future evidence syntheses.

Similarily, publication bias was assessed in only few reviews. This limited assessment suggests a potential gap in addressing this issue within the included reviews. Of the three studies that did analyze publication bias, findings were mixed. Holtedahl et al. [53] identified significant publication bias in the RCT subgroup, which could lead to an overestimation of treatment effects or effectiveness within these studies. Similarly, Zhao et al. [23] found publication bias specifically in outcomes related to male and female sex. This bias could misrepresent the true impact of gender on outcomes and lead to skewed interpretations in clinical decision-making. Overall, detection of publication bias in some of the included reviews raises concerns about the reliability, emphasizing the need for cautious interpretation and highlighting the importance of addressing publication bias systematically in future research to improve the reliability and validity of conclusions. To strengthen future evidence syntheses, systematic reviews and meta-analyses should routinely incorporate publication bias assessments using a combination of established methods such as Begg’s and Egger’s tests, funnel plot asymmetry, and sensitivity analyses. Applying these tools more consistently would help uncover hidden biases, support more balanced interpretations, and ultimately enhance the credibility and reproducibility of findings in this field.

The calculated CCA of 0.403 in this umbrella review indicates a moderate level of overlap among the included reviews, suggesting that a substantial proportion of the primary studies are shared across them. While moderate overlap can strengthen findings by demonstrating consistency in the evidence when similar conclusions are reached, it also risks overrepresentation of the same data, which may bias the overall analysis. To address this, particular attention was given to analyzing the meta-analyses within the included reviews. By focusing on these analyses, the umbrella review sought to synthesize evidence at a higher level, accounting for potential redundancy in primary study inclusion and ensuring a more balanced and comprehensive evaluation of the findings.

### Sociodemographic factors

Considering sociodemographic factors when developing a treatment plan is essential as these factors can significantly influence both surgical outcomes and the patient’s ability to adhere to postoperative protocols. The association of older age with an increased retear rate was consistently supported by multiple reviews [23, 36, 38, 42, 44, 46, 49, 52]. Reduced tendon-to-bone healing after RCR was observed with higher age, possibly due to lower local reserves of growth factors with increasing age, leading to reduced healing capacity [22, 54]. In addition, a disturbed arrangement of tendon-bone fibroblasts and reduced formation of collagenous fibrous tissue at the tendon-bone interface have been observed in older age, which reduces the stability of tendon healing [22]. However, the findings of this umbrella review also show that older age should not principally be considered a contraindication for surgical repair, as there was no clear evidence linking older age to inferior functional outcomes. Nevertheless, recognizing that older patients may face higher retear risks but still benefit functionally can guide surgical decisions and counseling.

Low BMD was also found to adversely affect tendon healing [23]. BMD is closely linked to age and typically decreases with advancing age [55]. This decline not only increases the risk of retear but also impacts the pull-out strength of anchors following RCR [56]. Greater bone density is critical for enhancing the strength of the tendon-to-bone attachment, and excessive osteoclastic activity, commonly observed in osteoporosis, may hinder bone ingrowth at the repair site, compromising the success of the surgical intervention [57]. However, although osteoporosis is more prevalent in women, no clear evidence was found to associate female sex with retear rates. Rather, factors such as diabetes and an increased BMI were identified as risk factors for retear. Since men generally have higher prevalence rates of diabetes and obesity [58, 59], this might explain why no clear evidence was found for a gender-specific difference in retear rates. Previous studies have shown that obesity is associated with poorer tendon quality [60, 61]. Additionally, obesity is linked to increased levels of proinflammatory cytokines and reactive oxygen species, which exacerbate inflammation and impair tendon healing. A high BMI also contributes to increased mechanical loads on the joints and greater pressure on tendons, further hindering the healing process [62]. In obese patients, both the operative time and the length of in-hospital stay may also be prolonged, which may negatively impact functional outcomes after RCR. Furthermore, diabetes has been shown to reduce fibrocartilage and tissue collagen formation at the tendon-bone interface following RCR, indicating that persistent hyperglycemia may inhibit the healing process [21].

#### Conflict

ing evidence was found regarding the influence of smoking on tendon healing. Animal studies have demonstrated that nicotine delays tendon-to-bone healing and remodeling of the supraspinatus tendon in rats after RCR, as it prolongs the inflammatory phase and reduces cell proliferation. While the meta-analysis by Fan et al. [20] supports these findings, the meta-analysis by Zhao et al. [23] did not identify a clear association between smoking and retear rates following RCR. However, a notable trend suggests that a generally healthy lifestyle was associated with a reduced risk of retear and improved functional outcomes following RCR.

In summary, although individual risk factors such as age, BMI, and diabetes were frequently identified as relevant in the included reviews, their potential interaction effects remain largely unexplored. Future studies should aim to quantitatively assess the interplay between multiple patient-specific factors using advanced statistical approaches such as multivariate logistic regression or structural equation modeling. Such methods would help to better understand the cumulative and possibly synergistic impact of these variables and support the development of personalized treatment algorithms.

### Genetic factors

Genes involved in the extracellular matrix (ECM), particularly those influencing collagen production, appeared to play a crucial role in tendon repair and remodeling. For instance, upregulation of MMP-3, which degrades ECM components, has been associated with reduced healing after RCR [47]. Dysregulated MMPs have also been implicated in cancer development [63], leading to investigations into MMP inhibition as a potential therapeutic strategy [64]. These advances may eventually have implications for improving tendon healing in RCR. Additionally, downregulation of COL3 has been linked to failed tendon healing [47], suggesting that COL3 is essential in the early healing process by providing cross-linking and scaffolding [25]. Strategies to stimulate COL3 expression, such as exposure to IL-17 A [65] or tocotrienol-rich fractions [66], could potentially enhance tendon repair after RCR. Another gene of interest is TNC, an ECM glycoprotein involved in modulating cell attachment and adhesion in newly formed tissue [67]. SNPs within the TNC-encoding gene have been associated with reduced healing after RCR [47].

Further, an SNP within the ESRRβ gene, which regulates apoptosis in hypoxic environments such as degenerated tendon tissue, has been associated with failure to heal [47]. This dysregulation likely results in increased apoptosis of musculoskeletal tissue. Additionally, upregulation of the growth factor BMP5 has been observed in healed rotator cuff tissue following RCR [47]. While BMP5 is primarily involved in bone and cartilage development, it appears to play a role in the physiological repair pathways of tendon healing.

While these genetic factors are unlikely to directly influence treatment decisions, they could serve as a foundation for developing medications to enhance tendon healing. However, further research will be necessary to explore this potential.

### Psychological factors

Psychosocial health is known to exert a significant impact on functional outcome, irrespective of the underlying pathology [68]. Although the psychological factors influencing outcomes were analyzed in the included reviews, no meta-analyses were identified, which limits the strength of the evidence. Among the psychological factors discussed in the included, high preoperative expectations were the sole factor found to positively impact functional outcome [34, 39, 48, 49]. However, a recent study by Karpinski et al. [69] revealed that expectations of patients regarding their operation differ from the surgeon’s assessment. Assessing and managing expectations, along with providing coping strategies, should therefore be considered essential for optimizing postoperative outcomes. Conversely, factors such as preoperative concerns, stress, pain catastrophizing, kinesiophobia, anxiety, and fear avoidance were associated with poorer postoperative outcomes. Similar findings have been reported in studies on other surgical procedures, which show that high preoperative anxiety levels are associated with an increased need for postoperative analgesics [24, 28]. These findings underscore the importance of evaluating psychological factors before surgery and acknowledging their impact on postoperative outcomes. However, effectively incorporating these factors into treatment strategies remains a challenge.

Evidence regarding the influence of depression on postoperative function was mixed. Depression has been linked to increased postoperative pain and functional limitations across various surgical procedures [26], as well as to a higher risk of postoperative infections [70, 71]. While the role of immune system dysregulation in depression is well documented [65], its specific impact on outcomes after RCR is poorly understood, emphasizing a significant gap in the current literature.

### Shoulder-specific factors

Clear evidence indicates that larger tear size negatively affects the retear rate. However, no evident association between tear size and functional outcomes was observed. Similarly, meta-analyses showed that factors such as the involvement of multiple tendons, tendon retraction, poor tissue quality, and the distance from the musculotendinous junction to the glenoid were associated with higher retear rates but did not show a clear influence on functional outcomes. These factors are likely interrelated, as is the duration of symptoms, which was also linked to higher retear rates. Patients should be informed that while these factors increase the risk of retear, the surgery can still yield favorable functional outcomes, as no direct correlation with functional improvement was identified.

The evidence regarding the role of fatty infiltration remains mixed. In fatty-infiltrated muscles, adipose tissue replaces internal muscle fibers, and the increased connective tissue and fibrosis reduce the elasticity and vitality of the tendon tissue [72]. Adipokines secreted by infiltrated adipocytes further contribute to chronic inflammation [27], potentially elevating the risk of retear. However, compensatory biological mechanisms during the repair process may mitigate these effects. Advances in surgical techniques and improved postoperative rehabilitation protocols may also reduce the influence of fatty infiltration on outcomes. To clarify its role, future studies employing standardized methodologies and better-controlled designs are required.

Additionally, evidence of the CSA to be associated with retear rates was reported. The compressive forces of the shoulder joint depend on the CSA, and shear forces increase as the CSA becomes larger. With a higher CSA, the supraspinatus muscle must exert additional force to maintain joint stability, potentially leading to overloading and an increased likelihood of rupture [73–75]. Similarly, the acromiohumeral interval was shown to influence retear rates. A shorter acromiohumeral interval may compromise glenohumeral mechanics, thereby increasing the risk of retear following rotator cuff repair and is associated with poorer rotator cuff quality [76].

### Limitations

This review is not without limitations. No statistical analysis of primary studies was conducted which is an underlying limitations of an umbrella review approach, which is designed to synthesize findings from existing systematic reviews and meta-analyses rather than to conduct new analyses on primary studies. Further, Multiple counting of primary studies that were included in multiple systematic reviews is possible. Hence, the most studied factors may be overrepresented. Additionally, only reviews and meta-analyses published after January 1st, 2012, were included to ensure that the synthesis reflects recent advancements in research methodologies and clinical practices while still including data from older primary studies. However, this date restriction could limit our analysis by potentially overlooking older reviews with robust findings. Nonetheless, by relying on recent reviews that incorporate both older and newer primary studies, we achieve a balanced synthesis that remains relevant to modern practices while retaining valuable historical data. In addition, comparison between studies was often difficult as often unclear definitions were used. Further, the results of the included reviews sometimes contradicted each other or were not clear regarding the impact of certain risk factors. Moreover, limitations in the methodological quality of the included studies and subsequently their evidence compromise the strength of recommendation of the present review. Only few of the publications contained meta-analyses as the diversity of the outcome measures and incomplete presentation of data in the primary studies often did not allow this. Further, due to the specific nature of the question, the included literature is limited to risk factors that can be measured preoperatively, so that other important factors such as surgical technique were not taken into account. Additionally, as with all reviews, there is a possibility that relevant studies were not identified and included in this analysis.

## Conclusion

This umbrella review highlights key factors influencing retear risk and functional outcomes after rotator cuff repair. Age consistently predicted higher retear rates, while high preoperative expectations were associated with improved outcomes. Reduced bone mineral density, elevated body mass index, and diabetes were linked to a higher risk of retear, while genetic markers such as MMP-3, COL3, and BMP5 were associated with tendon healing. Larger tears, involvement of multiple tendons, and poor tissue quality also contributed to increased retear risk without necessarily impacting functional outcomes. These findings underscore the importance of patient counseling, tailored surgical techniques, and targeted postoperative management, including addressing psychological factors and encouraging healthy lifestyles. However, uncertainties remain regarding factors like smoking and fatty infiltration, emphasizing the need for further research to enhance predictive models and develop more personalized treatment strategies.

## Electronic supplementary material

Below is the link to the electronic supplementary material.

Supplementary Material 1

Supplementary Material 2

## Data Availability

The datasets used and analysed during the current study are available from the corresponding author on reasonable request.

## Abbreviations

AMSTAR: A measurement tool to assess systematic reviews
BMP5: Bone morphogenetic protein 5
COL3: Collagen type III
CSA: Critical shoulder angle
ESRRβ: Estrogen related receptorβ
MMP3: Matrix metallopeptidase 3
RCR: Rotator cuff repair
SNP: Single nucleotide polymorphism
TNC: Tenascin C
WCB: Workers compensation board
