# Supplementary material for: Patient-specific risk factors for repair failure and poor functional outcome after rotator cuff repair - an umbrella review
Source: BMC Musculoskelet Disord. 2026 Feb 16;27:167. doi: 10.1186/s12891-025-08608-w (PMC12930581; doi:10.1186/s12891-025-08608-w)
Supplement: Supplementary file 1 — Supplementary Material 1 [file 12891_2025_8608_MOESM1_ESM.docx]

*Appendix 1: Search String*

("Rotator Cuff Injuries" OR "Rotator Cuff Tear*") AND ("Refixation" OR "Arthroscopy" OR "Open Surgery" OR "Operative surgical procedures" OR "surgery" OR "Surgical Procedures" OR "Operative") AND ("risk factor*" OR "Prognostic" OR "predictive" OR "inﬂuence" OR "outcome" OR "Rotator Cuff Retear*")
Article type: Systematic Review
Publication date: 2012/01/01 to 2022/05/14
