# Supplementary material for: Patient-specific risk factors for repair failure and poor functional outcome after rotator cuff repair - an umbrella review
Source: BMC Musculoskelet Disord. 2026 Feb 16;27:167. doi: 10.1186/s12891-025-08608-w (PMC12930581; doi:10.1186/s12891-025-08608-w)
Supplement: Supplementary file 2 — Supplementary Material 2 [file 12891_2025_8608_MOESM2_ESM.docx]

| Author Reference | Author systematic review  Reference | Altintas  et. al.  (2020) | Cimino  et al.  (2020) | Coronado  et al.  (2018) | Docter  et al.  (2019) | Fan  et al.  (2021) | Fermont  et al.  (2014) | Haunschild  et al.  (2021) | Holtedahl  et al.  (2021) | Kennedy  et al.  (2019) | Khair  et al.  (2016) | Kunze  et al.  (2020) | Lambers Heerspink  et al.  (2014) | Lapner  et al.  (2022) | Longo  et al.  (2021) | Lu  et al.  (2021) | McElvany  et al. (2014) | Mousley  et al.  (2021) | Panattoni  et al.  (2022) | Saccomanno  et al. (2016) | Santiago-Torres  et al.  (2014) | Sheean  et al.  (201) | Spennacchio  et al.  (2015) | Zhao  et al.  (2021) |
| --- | --- | --- | --- | --- | --- | --- | --- | --- | --- | --- | --- | --- | --- | --- | --- | --- | --- | --- | --- | --- | --- | --- | --- | --- |
|  |  |  |  |  |  |  |  |  |  |  |  |  |  |  |  |  | No further information about the 104 included studies given |  |  |  |  |  |  |  |
| Liem et al. (2008) | AJSM Outcome and structural integrity after arthroscopic rotator cuff repair using 2 rows of fixation: minimum 2-year follow-up. Am J Sports Med. 2006;34(12):1899-1905. | x |  |  |  |  |  |  |  |  |  |  |  |  |  |  |  |  |  |  |  |  |  |  |
| Dines et al. (2016) | Am J Orthop (Belle Mead NJ) Arthroscopic management of full-thickness rotator cuff tears in Major League Baseball pitchers: the lateralized footprint repair technique. Am J Orthop (Belle Mead NJ). 2016;45(3):128-133. | x |  |  |  |  |  |  |  |  |  |  |  |  |  |  |  |  |  |  |  |  |  |  |
| Ide et al. (2005) | AJSM Arthroscopic transtendon repair of partial-thickness articular-side tears of the rotator cuff: anatomical clinical study. Am J Sports Med. 2005;33(11):1672-1679. | x |  |  |  |  |  |  |  |  |  |  |  |  |  |  |  |  |  |  |  |  |  |  |
| Bhatia et al. (2015) | AJSM Two-year outcomes after arthroscopic rotator cuff repair in recreational athletes older than 70 years. Am J Sports Med. 2015;43(7):1737-1742. | x |  |  |  |  |  |  |  |  |  |  |  |  |  |  |  |  |  |  |  |  |  |  |
| Spencer (2010) | CORR Partial-thickness articular surface rotator cuff tears: an all-inside repair technique. Clin Orthop Relat Res. 2010;468(6):1514-1520. | x |  |  |  |  |  |  |  |  |  |  |  |  |  |  |  |  |  |  |  |  |  |  |
| Voos et al. (2007) | AJSM Outcomes of combined arthroscopic rotator cuff and labral repair. Am J Sports Med. 2007;35(7):1174-1179. | x |  |  |  |  |  |  |  |  |  |  |  |  |  |  |  |  |  |  |  |  |  |  |
| Antoni et al. (2016) | Orthop Traumatol Surg Res Return to recreational sport and clinical outcomes with at least 2 years follow-up after arthroscopic repair of rotator cuff tears. Orthop Traumatol Surg Res. 2016;102(5):563-567. | x |  |  |  |  |  |  |  |  |  |  |  |  |  |  |  |  |  |  |  |  |  |  |
| Tambe et al. (2009) | Int J Shoulder Surg Arthroscopic rotator cuff repair in competitive rugby players. Int J Shoulder Surg. 2009;3(1):8-12. | x |  |  |  |  |  |  |  |  |  |  |  |  |  |  |  |  |  |  |  |  |  |  |
| Simon et al. (2017) | Wilderness Environ Med Functional and sports-specific outcome after surgical repair of rotator cuff tears in rock climbers. Wilderness Environ Med. 2017;28(4):342-347. | x |  |  |  |  |  |  |  |  |  |  |  |  |  |  |  |  |  |  |  |  |  |  |
| Krishnan et al. (2008) | Arthroscopy Arthroscopic repair of full-thickness tears of the rotator cuff in patients younger than 40 years. Arthroscopy. 2008;24(3):324-328. | x |  |  |  |  |  |  |  |  |  |  |  |  |  |  |  |  |  |  |  |  |  |  |
| Burns and Snyder (2008) | JSES Arthroscopic rotator cuff repair in patients younger than fifty years of age. J Shoulder Elbow Surg. 2008;17(1):90-96. | x |  |  |  |  |  |  |  |  |  |  |  |  |  |  |  |  |  |  |  |  |  |  |
| Anderson et al. (2006) | AJSM Outcome and structural integrity after arthroscopic rotator cuff repair using 2 rows of fixation: minimum 2-year follow-up. Am J Sports Med. 2006;34(12):1899-1905. | x |  |  |  |  |  |  |  |  |  |  |  |  |  |  |  |  |  |  |  |  |  |  |
| Van Kleunen et al. (2012) | AJSM Return to high-level throwing after combination infraspinatus repair, slap repair, and release of glenohumeral internal rotation deficit. Am J Sports Med. 2012;40(11):2536-2541. | x |  |  |  |  |  |  |  |  |  |  |  |  |  |  |  |  |  |  |  |  |  |  |
| Franceschi et al. (2013) | Int Orthop Articular-sided rotator cuff tears: which is the best repair? A three-year prospective randomised controlled trial. Int Orthop. 2013;37(8):1487-1493. | x |  |  |  |  |  |  |  |  |  |  |  |  |  |  |  |  |  |  |  |  |  |  |
| Azzam et al. (2018) | AJSM Rotator cuff repair in adolescent athletes. Am J Sports Med. 2018;46(5):1084-1090. | x |  |  |  |  |  |  |  |  |  |  |  |  |  |  |  |  |  |  |  |  |  |  |
| Donohue et al. (2017) | Muscles Ligaments Tendons J Pre-operative corticosteroid injections improve functional outcomes in patients undergoing arthroscopic repair of high-grade partial-thickness rotator cuff tears Muscles Ligaments Tendons J, 7 (2017), pp. 34-39. |  | x |  |  |  |  |  |  |  |  |  |  |  |  |  |  |  |  |  |  |  |  |  |
| Tonotsuka et al. (2019) | Clin Orthop Surg Preoperative pain control in arthroscopic rotator cuff repair: Does it matter? Clin Orthop Surg, 11 (2019), pp. 192-199. |  | x |  |  |  |  |  |  |  |  |  |  |  |  |  |  |  |  |  |  |  |  |  |
| Traven et al. (2019) | Arthroscopy Preoperative shoulder injections are associated with increased risk of revision rotator cuff repair. Arthroscopy 2019;35:706-713. |  | x |  |  |  |  |  |  |  |  | x |  |  |  |  |  |  |  |  |  |  |  |  |
| Weber et al. (2019) | Arthroscopy Injections prior to rotator cuff repair are associated with increased rotator cuff revision rates. Arthroscopy 2019;35:717-724. |  | x |  |  |  |  |  |  |  |  | x |  |  |  |  |  |  |  |  |  |  |  |  |
| Agarwalla et al. (2019) | Arthroscopy Preoperative injections may be an iatrogenic cause of reoperation after arthroscopic rotator cuff repair Arthroscopy, 35 (2019), pp. 325-331. |  | x |  |  |  |  |  |  |  |  |  |  |  |  |  |  |  |  |  |  |  |  |  |
| Desai et al. (2019) | Arthroscopy Increasing numbers of shoulder corticosteroid injections within a year preoperatively may be associated with a higher rate of subsequent revision rotator cuff surgery. Arthroscopy 2019;35:45-50. |  | x |  |  |  |  |  |  |  |  | x |  |  |  |  |  |  |  |  |  |  |  |  |
| Baverel et al. (2017) | JSES Do corticosteroid injections compromise rotator cuff tendon healing after arthroscopic repair? JSES Open Access 2018;2:54-59. |  | x |  |  |  |  |  |  |  |  | x |  |  |  |  |  |  |  |  |  |  |  |  |
| Shin et al. (2016) | AJSM Efficacy of a subacromial corticosteroid injection for persistent pain after arthroscopic rotator cuff repair Am J Sports Med, 44 (2016), pp. 2231-2236. |  | x |  |  |  |  |  |  |  |  | x |  |  |  |  |  |  |  |  |  |  |  |  |
| Kim et al. (2019) | AJSM  Efficacy of a subacromial corticosteroid injection for persistent pain after arthroscopic rotator cuff repair Am J Sports Med, 44 (2016), pp. 2231-2236. |  | x |  |  |  |  |  |  |  |  |  |  |  |  |  |  |  |  |  |  |  |  |  |
| Lee et al (2019) | KSSTA  Intra-articular injection of steroids in the early postoperative period does not have an adverse effect on the clinical outcomes and the re-tear rate after arthroscopic rotator cuff repair Knee Surg Sports Traumatol Arthrosc, 27 (2019), pp. 3912-3919. |  | x |  |  |  |  |  |  |  |  | x |  |  |  |  |  |  |  |  |  |  |  |  |
| Kim and Jung (2018) | AJSM  An intra-articular steroid injection at 6 weeks postoperatively for shoulder stiffness after arthroscopic rotator cuff repair does not affect repair integrity Am J Sports Med, 46 (2018), pp. 2192-2202. |  | x |  |  |  |  |  |  |  |  | x |  |  |  |  |  |  |  |  |  |  |  |  |
| Barlow et al. (2016) | JSES What factors are predictors of emotional health in patients with full-thickness rotator cuff tears? J Shoulder Elbow Surg. 2016;25:1769 – 1773. |  |  | x |  |  |  |  |  | x |  |  |  |  |  |  |  |  | x |  |  |  |  |  |
| Cho et al. (2013) | JSES The impact of depression and anxiety on self-assessed pain, disability, and quality of life in patients scheduled for rotator cuff repair. J Shoulder Elbow Surg. 2013;22:1160 – 1166. |  |  | x |  |  |  |  |  | x |  |  |  |  |  |  |  |  | x |  |  |  |  |  |
| Cho et al. (2015) | Clin Orthop Relat Does rotator cuff repair improve psychologic status and quality of life in patients with rotator cuff tear? Clin Orthop Relat Res. 2015;473:3494 – 3500. |  |  | x |  |  |  |  |  | x |  |  |  |  |  |  |  |  | x |  |  |  |  |  |
| Henn et al. (2007) | JBJS Patients ’ pre-operative expectations predict the outcome of rotator cuff repair. J Bone Joint Surg Am. 2007;89:1913 – 1919. |  |  | x |  |  |  |  |  | x |  |  | x |  |  |  |  |  | x | x |  |  |  |  |
| Oh et al. (2012) | JSES Effect of expectations and concerns in rotator cuff disorders and correlations with preoperative patient characteristics. J Shoulder Elbow Surg. 2012;21:715 – 721. |  |  | x |  |  |  |  |  | x |  |  |  |  |  |  |  |  | x | x |  |  |  |  |
| Piitulainen et al. (2012) | Disabil Rehabil  The relationship between functional disability and health-related qual- ity of life in patients with a rotator cuff tear. Disabil Rehabil. 2012;34:2071 – 2075. |  |  | x |  |  |  |  |  | x |  |  |  |  |  |  |  |  | x |  |  |  |  |  |
| Potter et al. (2015) | Clin Orthop Relat One-year patient-reported outcomes after arthroscopic ro- tator cuff repair do not correlate with mild to moderate psycho- logical distress. Clin Orthop Relat Res. 2015;473:3501 – 3510. |  |  | x |  |  |  |  |  | x |  |  |  |  |  |  |  |  | x |  |  |  |  |  |
| Tashjian et al. (2006) | JBJS Effect of medical comorbidity on self-assessed pain, function, and general health status after rotator cuff repair. J Bone Joint Surg Am. 2006;88:536 – 540. |  |  | x |  |  |  |  |  |  |  |  | x |  |  |  |  |  |  | x |  |  |  |  |
| Woollard et al. (2016) | Disabil Rehabil  The ability of preoperative factors to predict patient-reported disability following surgery for rotator cuff pathology. Disabil Rehabil. 2016:1 – 10. |  |  | x |  |  |  |  |  |  |  |  |  |  |  |  |  |  | x |  |  |  |  |  |
| Wylie et al. (2016) | J Bone Joint Surg Am  Mental health has a stronger association with patient-reported shoulder pain and function than tear size in patients with full-thickness rotator cuff tears. J Bone Joint Surg Am. 2016;98:251 – 256. |  |  | x |  |  |  |  |  | x |  |  |  |  |  |  |  |  | x |  |  |  |  |  |
| Moor et al. (2013) | Bone Joint J  Is there an association between the individual anatomy of the scapula and the development of rotator cuff tears or osteoarthritis of the glenohumeral joint? A radiological study of the critical shoulder angle Bone Joint J, 95-B (2013), pp. 935-941. |  |  |  | x |  |  |  |  |  |  |  |  |  |  |  |  |  |  |  |  |  |  |  |
| Moor et al. (2014) | JSES Relationship of individual scapular anatomy and degenerative rotator cuff tears J Shoulder Elbow Surg, 23 (2014), pp. 536-541. |  |  |  | x |  |  |  |  |  |  |  |  |  |  |  |  |  |  |  |  |  |  |  |
| Pandey et al. (2016) | JSES Does scapular morphology affect the integrity of the rotator cuff? J Shoulder Elbow Surg, 25 (2016), pp. 413-421. |  |  |  | x |  |  |  |  |  |  |  |  |  |  |  |  |  |  |  |  |  |  |  |
| Spiegl et al. (2016) | KSSTA The critical shoulder angle is associated with rotator cuff tears and shoulder osteoarthritis and is better assessed with radiographs over MRI Knee Surg Sports Traumatol Arthrosc, 24 (2016), pp. 2244-2251. |  |  |  | x |  |  |  |  |  |  |  |  |  |  |  |  |  |  |  |  |  |  |  |
| Chalmers et al. (2017) | CORR Does the critical shoulder angle correlate with rotator cuff tear progression? Clin Orthop Relat Res, 475 (2017), pp. 1608-1617. |  |  |  | x |  |  |  |  |  |  |  |  |  |  |  |  |  |  |  |  |  |  |  |
| Heuberer et al. (2017) | BMC Musculoskelet Disord Critical shoulder angle combined with age predict five shoulder pathologies: A retrospective analysis of 1000 cases BMC Musculoskelet Disord. 2017 Jun 15;18(1):259. |  |  |  | x |  |  |  |  |  |  |  |  |  |  |  |  |  |  |  |  |  |  |  |
| Shingawa et al. (2018) | JSES Critical shoulder angle in an East Asian population: Correlation to the incidence of rotator cuff tear and glenohumeral osteoarthritis J Shoulder Elbow Surg, 27 (2018), pp. 1602-1606. |  |  |  | x |  |  |  |  |  |  |  |  |  |  |  |  |  |  |  |  |  |  |  |
| Scheiderer et al. (2018) | Arthroscopy Higher critical shoulder angle and acromion index are associated with increased retear risk after isolated supraspinatus tendon repair at short-term follow up Arthroscopy, 34 (2018), pp. 2748-2754. |  |  |  | x |  |  |  |  |  |  |  |  |  |  |  |  |  |  |  |  | x |  |  |
| Garcia et al. (2017) | JSES Higher critical shoulder angle increases the risk of retear after rotator cuff repair J Shoulder Elbow Surg, 26 (2017), pp. 241-245. |  |  |  | x |  |  |  |  |  |  |  |  |  |  |  |  |  |  |  |  | x |  |  |
| Kirsch et al. (2017) | OJSM Is there an association between the “critical shoulder angle” and clinical outcome after rotator cuff repair? Orthop J Sports Med, 5 (2017) 232596711770212. |  |  |  | x |  |  |  |  |  |  |  |  |  |  |  |  |  |  |  |  | x |  |  |
| Lee et al. (2017) | AJSM Critical shoulder angle and acromial index do not influence 24-month functional outcome after arthroscopic rotator cuff repair Am J Sports Med, 45 (2017), pp. 2989-2994. |  |  |  | x |  |  |  |  |  |  |  |  |  |  |  |  |  |  |  |  | x |  |  |
| Li et al. (2018) | AJSM Large critical shoulder angle has higher risk of tendon retear after arthroscopic rotator cuff repair Am J Sports Med, 46 (2018), pp. 1892-1900. |  |  |  | x |  |  |  |  |  |  |  |  |  |  |  |  |  |  |  |  | x |  |  |
| Mallon et al. (2004) | JSES The impact of preoperative smoking habits on the results of rotator cuff repair. J Shoulder Elbow Surg 13:129–132. |  |  |  |  | x |  |  |  |  |  |  |  |  |  |  |  |  |  | x | x |  |  |  |
| Djahangiri et al. (2013) | JSES Outcome of single-tendon rotator cuff repair in patients aged older than 65 years. J Shoulder Elbow Surg 22:45–51. |  |  |  |  | x |  |  |  |  |  |  |  |  |  |  |  |  |  | x | x |  |  |  |
| Neyton et al. (2013) | Arthroscopy Arthroscopic suture-bridge repair for small to medium size supraspinatus tear: healing rate and retear pattern. Arthroscopy. 2013;29(1):10-17. |  |  |  |  | x |  |  |  |  |  |  |  |  |  |  |  |  |  |  | x |  |  |  |
| Kukkonen et al. (2014) | Scand J Med Sci Sports Smoking and operative treatment of rotator cuff tear Scand J Med Sci Sports, 24 (2014), pp. 400-403. |  |  |  |  | x |  |  |  |  |  |  |  |  |  |  |  |  |  |  |  |  |  |  |
| Saraswat et al. (2015) | AJSM Functional outcomes and health-related quality of life after surgical repair of full-thickness rotator cuff tears using a mini-open technique: a concise 10-year follow-up of a previous report Am J Sports Med, 43 (2015), pp. 2794-2799. |  |  |  |  | x |  |  |  |  |  |  |  |  |  |  |  |  |  |  |  |  |  |  |
| Cuff et al. (2016) | Arthroscopy Evaluation of factors affecting acute postoperative pain levels after arthroscopic rotator cuff repair Arthroscopy, 32 (2016), pp. 1231-1236. |  |  |  |  | x |  |  |  |  |  |  |  |  |  |  |  |  |  |  |  |  |  |  |
| Mahure et al. (2016) | Arthroscopy The incidence of subsequent surgery after outpatient arthroscopic rotator cuff repair Arthroscopy, 32 (2016), pp. 1531-1541. |  |  |  |  | x |  |  |  |  |  |  |  |  |  |  |  |  |  |  |  |  |  |  |
| Park et al. (2018) | AJSM Effect of smoking on healing failure after rotator cuff repair Am J Sports Med, 46 (2018), pp. 2960-2968. |  |  |  |  | x |  |  |  |  |  |  |  |  |  |  |  |  |  |  |  |  |  |  |
| Nicholson et al. (2019) | Bone Joint J Cost-effectiveness and satisfaction following arthroscopic rotator cuff repair: does age matter? Bone Joint J, 101-b (2019), pp. 860-866. |  |  |  |  | x |  |  |  |  |  |  |  |  |  |  |  |  |  |  |  |  |  |  |
| Baumgarten et al. (2020) | JSES Do patients who smoke tobacco have success with primary arthroscopic rotator cuff repair? A comparison with nonsmokers J Shoulder Elbow Surg, 29 (2020), pp. 1650-1655. |  |  |  |  | x |  |  |  |  |  |  |  |  |  |  |  |  |  |  |  |  |  |  |
| O'Donnell et al. (2020) | Arthroscopy The effect of patient characteristics and comorbidities on the rate of revision rotator cuff repair Arthroscopy, 36 (2020), pp. 2380-2388. |  |  |  |  | x |  |  |  |  |  |  |  |  |  |  |  |  |  |  |  |  |  |  |
| Lobo-Escolar et al. (2021) | JSES Risk factors for symptomatic retears after arthroscopic repair of full-thickness rotator cuff tears J Shoulder Elbow Surg, 30 (2021), pp. 27-33. |  |  |  |  | x |  |  |  |  |  |  |  |  |  |  |  |  |  |  |  |  | x |  |
| Zabrzynski et al. (2021) | J Clin Med The impact of smoking on clinical results following the rotator cuff and biceps tendon complex arthroscopic surgery J Clin Med, 10 (2021), p. 599. |  |  |  |  | x |  |  |  |  |  |  |  |  |  |  |  |  |  |  |  |  |  |  |
| Boileau et al. (2005) | JBJS Arthroscopic repair of full-thickness tears of the supraspinatus: Does the tendon really heal? J Bone Joint Surg Am 87:1229–1240. |  |  |  |  |  | x |  |  |  |  |  |  |  |  |  |  |  |  | x |  |  |  |  |
| Cho and Rhee (2009) | Clin Orthop Surg   The factors affecting the  clinical outcome and integrity of arthroscopi- cally repaired rotator cuff tears of the shoulder.  Clin Orthop Surg. 2009;1:96-104. |  |  |  |  |  | x |  |  |  | x |  |  |  |  |  |  |  |  | x |  |  |  |  |
| Chung et al. (2013) | Am J Sports Med Arthroscopic repair of massive rotator cuff tears: outcome and analysis of factors associated with healing failure or poor ostoperative function.  Am J Sports Med. 2013;41:1674-1683. |  |  |  |  |  | x |  |  |  |  |  |  |  |  | x |  |  |  | x |  |  |  |  |
| Chung et al. (2011) | Am J Sports Med Factors affecting rotator cuff healing after arthroscopic repair: osteoporosis as one of the independent risk factors. Am J Sports Med. 2011;39:2099-2107. |  |  |  |  |  | x |  |  |  |  |  |  |  |  | x |  |  |  | x |  |  |  | x |
| Chung et al. (2012) | Am J Sports Med Quality of life after arthroscopic rotator cuff repair: evaluation using SF-36 and an analysis of affecting clinical factors. Am J Sports Med. 2012;40:631-639. |  |  |  |  |  | x |  |  | x |  |  |  |  |  |  |  |  |  | x |  |  |  |  |
| Gulotta et al. (2011) | JSES  Prospective evaluation of arthroscopic rotator cuff repairs at 5 years: part II—prognostic factors for clinical and radiographic outcomes J Shoulder Elbow Surg, 20 (2011), pp. 941-946. |  |  |  |  |  | x |  |  |  |  |  | x |  |  |  |  |  |  | x |  |  | x |  |
| Lapner et al. (2010) | CORR Does the upward migration index predict function and quality of life in arthroscopic rotator cuff repair? Clin Orthop Relat Res.  2010;468:3063-3069. |  |  |  |  |  | x |  |  |  |  |  |  |  |  |  |  |  |  | x |  |  |  |  |
| Manaka et al. (2011) | CORR Functional recovery period after  arthroscopic rotator cuff repair: is it predict- able before surgery? Clin Orthop Relat Res.  2011;469:1660-1666. |  |  |  |  |  | x |  |  |  |  |  |  |  |  |  |  |  |  |  |  |  |  |  |
| Warrender et al. (2011) | JSES Outcomes of arthroscopic rotator cuff repairs in obese patients. J Shoulder Elbow Surg. 2011;20:961-967. |  |  |  |  |  | x |  |  |  |  |  |  |  |  |  |  |  |  | x |  |  |  |  |
| Wessel et al. (2007) | Ned Tijdschr Orthop Artroscopisch rotator cuffrepair nader  geanalyseerd: een prospectieve studie bij 70  patiënten met een rotator cufflaesie. Ned Tijd- schr Orthop. 2007;4:137-142. |  |  |  |  |  | x |  |  |  |  |  |  |  |  |  |  |  |  |  |  |  |  |  |
| Aagaard et al. (2020) | Eur J Trauma Emerg Surg Return to work after early repair of acute traumatic rotator cuff tears. Eur J Trauma Emerg Surg. 2020;46(4):817-823. |  |  |  |  |  |  | x |  |  |  |  |  |  |  |  |  |  |  |  |  |  |  |  |
| Baysal et al. (2005) | AJSM Functional outcome and health-related quality of life after surgical repair of full-thickness rotator cuff tear using a mini-open technique. Am J Sports Med. 2005;33(9):1346-1355. |  |  |  |  |  |  | x |  |  |  |  |  |  |  |  |  |  |  |  |  |  |  |  |
| Bhatia et al. (2010) | Arthroscopy Early return to work in workers’ compensation patients after arthroscopic full-thickness rotator cuff repair. Arthroscopy. 2010;26(8):1027-1034, e1123. |  |  |  |  |  |  | x |  |  |  |  |  |  |  |  |  |  |  |  |  |  |  |  |
| Didden et al. (2010) | Acta Orthop Belg  The impact of the Belgian workers’ compensation system on return to work after rotator cuff surgery. Acta Orthop Belg. 2010;76(5):592-597. |  |  |  |  |  |  | x |  |  |  |  |  |  |  |  |  |  |  |  |  |  |  |  |
| Gowd et al. (2019) | Orthop J Sports Med Preoperative mental health scores and achieving patient acceptable symptom state are predictive of return to work after arthroscopic rotator cuff repair. Orthop J Sports Med. 2019;7(10):2325967119878415. |  |  |  |  |  |  | x |  |  |  |  |  |  |  |  |  |  |  |  |  |  |  |  |
| Hawkins et al. (1999) | JSES Surgical treatment of full-thickness rotator cuff tears in patients 40 years of age or younger. J Shoulder Elbow Surg. 1999;8(3):259-265. |  |  |  |  |  |  | x |  |  |  |  |  |  |  |  |  |  |  |  |  |  |  |  |
| Denard et al. (2015) | AJSM Pseudoparalysis from a massive rotator cuff tear is reliably reversed with an arthroscopic rotator cuff repair in patients without preoperative glenohumeral arthritis. Am J Sports Med. 2015;43(10):2373-2378. |  |  |  |  |  |  | x |  |  |  |  |  |  |  |  |  |  |  |  |  |  |  |  |
| Lin et al. (2012) | Acta Orthop Belg Rotator cuff tears in patients younger than 50 years of age. Acta Orthop Belg. 2012;78(5):592-596. |  |  |  |  |  |  | x |  |  |  |  |  |  |  |  |  |  |  |  |  |  |  |  |
| Nové-Josserand et al. (2011) | Orthop Traumatol Surg Res Occupational outcome after surgery in patients with a rotator cuff tear due to a work-related injury or occupational disease: a series of 262 cases. Orthop Traumatol Surg Res. 2011;97(4):361-366. |  |  |  |  |  |  | x |  |  |  |  |  |  |  |  |  |  |  |  |  |  |  |  |
| Noyes et al. (2019) | Arthroscopy Functional outcome and healing with a load-sharing rip-stop repair compared with a single-row repair for large and massive rotator cuff tears. Arthroscopy. 2019;35(8):2295-2300. |  |  |  |  |  |  | x |  |  |  |  |  |  |  |  |  |  |  |  |  |  |  |  |
| Razmjou et al. (2017) | JSES The effect of expedited rotator cuff surgery in injured workers: a case-control study. J Shoulder Elbow Surg. 2017;26(7):1196-1202. |  |  |  |  |  |  | x |  |  |  |  |  |  |  |  |  |  |  |  |  |  |  |  |
| Imai et al. (2019) | Prog Rehabil Med Factors affecting return to work in patients undergoing arthroscopic rotator cuff repair. Prog Rehabil Med. 2019;4:20190006. |  |  |  |  |  |  | x |  |  |  |  |  |  |  |  |  |  |  |  |  |  |  |  |
| Misamore et al. (1995) | JBJS Am Repair of the rotator cuff: a comparison of results in two populations of patients. J Bone Joint Surg Am. 1995;77(9):1335-1339. |  |  |  |  |  |  | x |  |  |  |  |  |  |  |  |  |  |  |  |  |  |  |  |
| Bryant et al. (2016) | JSES A randomized clinical trial to compare the effectiveness of rotator cuff repair with or without augmentation using porcine small intestine submucosa for patients with moderate to large rotator cuff tears: A pilot study J Shoulder Elbow Surg, 25 (2016), pp. 1623-1633. |  |  |  |  |  |  |  | x |  |  |  |  |  |  |  |  |  |  |  |  |  |  |  |
| Burks et al. (2009) | AJSM  A prospective randomized clinical trial comparing arthroscopic single- and double-row rotator cuff repair: Magnetic resonance imaging and early clinical evaluation. Am J Sports Med 2009;37:674-682. |  |  |  |  |  |  |  | x |  |  |  |  | x | x |  |  |  |  |  |  |  |  |  |
| Carr et al. (2015) | Health Technol Assess  Clinical effectiveness and cost-effectiveness of open and arthroscopic rotator cuff repair [the UK rotator cuff surgery (UKUFF) randomised trial] Health Technol Assess, 19 (2015), pp. 1-217. |  |  |  |  |  |  |  | x |  |  |  |  |  |  |  |  |  |  |  |  |  |  |  |
| Castricini et al. (2011) | AJSM Platelet-rich plasma augmentation for arthroscopic rotator cuff repair: a randomized controlled trial. Am J Sports Med. 2011;39(2):258–265. doi: 10.1177/0363546510390780. |  |  |  |  |  |  |  | x |  |  |  |  |  | x |  |  |  |  |  |  |  |  |  |
| Dezaly et al. (2011) | Orthop Traumatol Surg Res Arthroscopic treatment of rotator cuff tear in the over-60s: Repair is preferable to isolated acromioplasty-tenotomy in the short term Orthop Traumatol Surg Res, 97 (6 Suppl) (2011), pp. S125-S130. |  |  |  |  |  |  |  | x |  |  |  |  |  |  |  |  |  |  |  |  |  |  |  |
| Flury et al. (2016) | AJSM Does pure platelet-rich plasma affect postoperative clinical outcomes after arthroscopic rotator Cuff repair? A randomized controlled trial. Am J Sports Med. 2016;44(8):2136–2146. |  |  |  |  |  |  |  | x |  |  |  |  |  |  |  |  |  |  |  |  |  |  |  |
| Gumina et al. (2012) | Curr Med Res Opin Arginine L-alpha-ketoglutarate, methylsulfonylmethane, hydrolyzed type I collagen and bromelain in rotator cuff tear repair: a prospective randomized study. Curr Med Res Opin. 2012;28(11):1767–1774. |  |  |  |  |  |  |  | x |  |  |  |  |  |  |  |  |  |  |  |  |  |  |  |
| Gumina et al. (2012) | JBJS Use of platelet-leukocyte membrane in arthroscopic repair of large rotator cuff tears: a prospective randomized study. J Bone Joint Surg Am. 2012;94(15):1345–1352. |  |  |  |  |  |  |  | x |  |  |  |  |  |  |  |  |  |  |  |  |  |  |  |
| Lambers Heerspink et al. (2015) | JSES Comparing surgical repair with conservative treatment for degenerative rotator cuff tears: a randomized controlled trial. J Shoulder Elb Surg. 2015;24(8):1274–1281. |  |  |  |  |  |  |  | x |  |  |  |  |  |  |  |  |  |  |  |  |  |  |  |
| Heuberer et al. (2020) | AJSM  Delaminated rotator cuff tears showed lower short-term retear rates after arthroscopic double-layer repair versus bursal layer-only repair: A randomized controlled trial Am J Sports Med, 48 (2020), pp. 689-696. |  |  |  |  |  |  |  | x |  |  |  |  |  |  |  |  |  |  |  |  |  |  |  |
| Jacquot et al. (2014) | Orthop Traumatol Surg Res Is rotator cuff repair appropriate in patients older than 60 years of age? Prospective, randomised trial in 103 patients with a mean four-year follow-up. Orthop Traumatol Surg Res. 2014;100(6 Suppl):S333–S338. |  |  |  |  |  |  |  | x |  |  |  |  |  |  |  |  |  |  |  |  |  |  |  |
| Jenssen et al. (2018) | Arthroscopy  No functional difference between three and six weeks of immobilization after arthroscopic rotator Cuff repair: a prospective randomized controlled non-inferiority trial. Arthroscopy. 2018;34(10):2765–2774. |  |  |  |  |  |  |  | x |  |  |  |  |  | x |  |  |  |  |  |  |  |  |  |
| Jo et al. (2013) | AJSM Platelet-rich plasma for arthroscopic repair of large to massive rotator cuff tears: a randomized, single-blind, parallel-group trial. Am J Sports Med. 2013;41(10):2240–2248. |  |  |  |  |  |  |  | x |  |  |  |  |  | x |  |  |  |  |  |  |  |  |  |
| Jo et al. (2015) | AJSM Platelet-rich plasma for arthroscopic repair of medium to large rotator cuff tears. Am J Sports Med 2015;43:2102-2110. |  |  |  |  |  |  |  | x |  |  |  |  |  |  |  |  |  |  |  |  |  |  |  |
| Keener et al. (2014) | JBJS  Rehabilitation following arthroscopic rotator cuff repair: a prospective randomized trial of immobilization compared with early motion. J Bone Joint Surg Am. 2014;96(1):11–19. |  |  |  |  |  |  |  | x |  |  |  |  |  | x |  |  |  |  |  |  |  |  |  |
| Koh et al. (2011) | Arthroscopy  Prospective randomized clinical trial of single- versus double-row suture anchor repair in 2- to 4-cm rotator cuff tears: Clinical and magnetic resonance imaging results. Arthroscopy 2011;27:453-462. |  |  |  |  |  |  |  | x |  |  |  |  | x | x |  |  |  |  |  |  |  |  |  |
| Kukkonen et al. (2015) | JBJS Treatment of nontraumatic rotator cuff tears J Bone Joint Surg, 97 (2015), pp. 1729-1737. |  |  |  |  |  |  |  | x |  |  |  |  |  |  |  |  |  |  |  |  |  |  |  |
| Lapner et al. (2012) | JBJS A multicenter randomized controlled trial comparing single-row with double-row ﬁ xation in arthroscopic rotator cuff repair. J Bone Joint Surg Am 2012;94:1249-1257. |  |  |  |  |  |  |  | x |  |  |  |  | x | x |  |  |  |  | x |  |  |  |  |
| Ma et al. (2012) | Arthroscopy Clinical outcome and imaging of arthroscopic single-row and double-row rotator cuff repair: A prospective randomized trial. Arthroscopy 2012;28:16-24. |  |  |  |  |  |  |  | x |  |  |  |  | x | x |  |  |  |  |  |  |  |  |  |
| Ma et al. (2019) | AJSM Does arthroscopic suture-spanning augmentation of single-row repair reduce the retear rate of massive rotator cuff tear ? Am J Sports Med 2019;47:1420-1426. |  |  |  |  |  |  |  | x |  |  |  |  |  |  |  |  |  |  |  |  |  |  |  |
| Malavolta et al. (2014) | AJSM Platelet-rich plasma in rotator cuff repair: A prospective randomized study. Am J Sports Med 2014;42:2446-2454. |  |  |  |  |  |  |  | x |  |  |  |  |  |  |  |  |  |  |  |  |  |  |  |
| Mazzocca et al. (2017) | Arthroscopy  The effect of early range of motion on quality of life, clinical outcome, and repair integrity after arthroscopic rotator Cuff repair. Arthroscopy. 2017;33(6):1138–1148. |  |  |  |  |  |  |  | x |  |  |  |  |  | x |  |  |  |  |  |  |  |  |  |
| Moosmayer et al. (2010) | JBJS Br Comparison between surgery and physiotherapy in the treatment of small and medium-sized tears of the rotator cuff: A randomised controlled study of 103 patients with one-year follow-up J Bone Joint Surg Br, 92 (2010), pp. 83-91. |  |  |  |  |  |  |  | x |  |  |  |  |  |  |  |  |  |  |  |  |  |  |  |
| Oh et al. (2018) | JSES Effect of recombinant human growth hormone on rotator cuff healing after arthroscopic repair: preliminary result of a multicenter, prospective, randomized, open-label blinded end point clinical exploratory trial J Shoulder Elbow Surg, 27 (2018), pp. 777-785. |  |  |  |  |  |  |  | x |  |  |  |  |  |  |  |  |  |  |  |  |  |  |  |
| Oh et al. (2020) | AJSM Maximum bridging suture tension provides better clinical outcomes in transosseous-equivalent rotator cuff repair: A clinical, prospective randomized comparative study Am J Sports Med, 48 (2020), pp. 2129-2136. |  |  |  |  |  |  |  | x |  |  |  |  |  |  |  |  |  |  |  |  |  |  |  |
| Randelli et al. (2011) | JSES Platelet-rich plasma in arthroscopic rotator cuff repair: A prospective Rct study, 2-year follow-up. J Shoulder Elbow Surg 2011;20:518-528. |  |  |  |  |  |  |  | x |  |  |  |  |  | x |  |  |  |  |  |  |  |  |  |
| Rodeo et al. (2012) | AJSM The effect of platelet-rich fibrin matrix on rotator cuff tendon healing: a prospective, randomized clinical study. Am J Sports Med. 2012;40(6):1234–1241. |  |  |  |  |  |  |  | x |  |  |  |  |  | x |  |  |  |  |  |  |  |  |  |
| Ruiz-Moneo et al. (2013) | Arthroscopy Plasma rich in growth factors in arthroscopic rotator cuff repair: a randomized, double-blind, controlled clinical trial. Arthroscopy : the journal of arthroscopic & related surgery : official publication of the Arthroscopy Association of North America and the International Arthroscopy Association. 2013;29(1):2–9. |  |  |  |  |  |  |  | x |  |  |  |  |  |  |  |  |  |  |  |  |  |  |  |
| Shin et al. (2012) | Arthroscopy The efficacy of acromioplasty in the arthroscopic repair of small- to medium-sized rotator cuff tears without acromial spur: prospective comparative study. Arthroscopy. 2012;28(5):628–635. |  |  |  |  |  |  |  | x |  |  |  |  |  |  |  |  |  |  |  |  |  |  |  |
| van der Zwaal (2013) | Arthroscopy  Clinical outcome in all-arthroscopic versus mini-open rotator cuff repair in small to medium-sized tears: a randomized controlled trial in 100 patients with 1-year follow-up. Arthroscopy. 2013;29(2):266–273. |  |  |  |  |  |  |  | x |  |  |  |  |  | x |  |  |  |  |  |  |  |  |  |
| Walsh et al. (2018) | JSES Platelet-rich plasma in fibrin matrix to augment rotator cuff repair: a prospective, single-blinded, randomized study with 2-year follow-up. J Shoulder Elb Surg. 2018;27(9):1553–1563. |  |  |  |  |  |  |  | x |  |  |  |  |  | x |  |  |  |  |  |  |  |  |  |
| Yamakado (2019) | Arthroscopy  A prospective randomized trial comparing suture bridge and medially based single-row rotator cuff repair in medium-sized supraspinatus tears. Arthroscopy 2019;35:2803-2813. |  |  |  |  |  |  |  | x |  |  |  |  | x |  |  |  |  |  |  |  |  |  |  |
| Zhang et al. (2016) | Acta Orthop Traumatol Turc The effect of platelet-rich plasma on arthroscopic double-row rotator cuff repair: a clinical study with 12-month follow-up. Acta Orthop Traumatol Turc. 2016;50(2):191–197. |  |  |  |  |  |  |  | x |  |  |  |  |  |  |  |  |  |  |  |  |  |  |  |
| Zumstein et al. (2016) | JSES Use of platelet- and leucocyte-rich ﬁ brin (L-PRF) does not affect late rotator cuff tendon healing: A prospective randomized controlled study. J Shoulder Elbow Surg 2016;25:2-11. |  |  |  |  |  |  |  | x |  |  |  |  |  | x |  |  |  |  |  |  |  |  |  |
| Bayle et al. (2017) | AOTS No difference in outcome for open versus arthroscopic rotator cuff repair: A prospective comparative trial. Arch Orthop Trauma Surg, 137 (2017), pp. 1707-1712. |  |  |  |  |  |  |  | x |  |  |  |  |  |  |  |  |  |  |  |  |  |  |  |
| Boyer et al. (2013) | Arthroscopy Arthroscopic double-row cuff repair with suture-bridging: A structural and functional comparison of two techniques. Arthroscopy, 23 (2015), pp. 478-486. |  |  |  |  |  |  |  | x |  |  |  |  |  |  |  |  |  |  |  |  |  |  |  |
| Jo et al. (2011) | AJSM Does platelet-rich plasma accelerate recovery after rotator cuff repair? A prospective cohort study. Am J Sports Med, 39 (2011), pp. 2082-2090. |  |  |  |  |  |  |  | x |  |  |  |  |  |  |  |  |  |  |  |  |  |  |  |
| Kim et al. (2012) | AJSM Repair integrity and functional outcome after arthroscopic rotator cuff repair: Double-row versus suture-bridge technique. Am J Sports Med 2012;40:294-299. |  |  |  |  |  |  |  | x |  |  |  |  |  |  |  |  |  |  |  |  |  |  |  |
| Kim et al. (2013) | AJSM  Comparison of repair integrity and functional outcomes for 3 arthroscopic suture bridge rotator cuff repair techniques. Am J Sports Med 2013;41:271-277. |  |  |  |  |  |  |  | x |  |  |  |  |  |  |  |  |  |  |  |  |  |  |  |
| Kim et al. (2018) | J Orthop Traum Surg Res Clinical outcomes and repair integrity of arthroscopic rotator cuff repair using suture-bridge technique with or without medial tying: Prospective comparative study. J Orthop Traum Surg Res 2018;13:1-8. |  |  |  |  |  |  |  | x |  |  |  |  |  |  |  |  |  |  |  |  |  |  |  |
| Kim et al. (2015) | AJSM Conventional en masse repair versus separate double-layer double-row repair for the treatment of delaminated rotator cuff tears. Am J Sports Med 2015;44:1146-1152. |  |  |  |  |  |  |  | x |  |  |  |  |  |  |  |  |  |  |  |  |  |  |  |
| Bonnevialle et al. (2015) | JSES Does microvascularization of the footprint play a role in rotator cuff healing of the shoulder?. J Shoulder Elbow Surg, 24 (2015), pp. 1257-1262. |  |  |  |  |  |  |  | x |  |  |  |  |  |  |  |  |  |  |  |  |  |  |  |
| Gladstone et al. (2007) | AJSM Fatty infiltration and atrophy of the rotator cuff do not improve after rotator cuff repair and correlate with poor functional outcome. Am J Sports Med. 2007; 35(5): 719-728. |  |  |  |  |  |  |  | x |  | x |  | x |  |  |  |  |  |  | x |  |  |  |  |
| Klepps et al. (2004) | AJSM Prospective evaluation of the effect of rotator cuff integrity on the outcome of open rotator cuff repairs. Am J Sports Med, 32 (2004), pp. 1716-1722. |  |  |  |  |  |  |  | x |  |  |  |  |  |  |  |  |  |  |  |  |  |  |  |
| Levy et al. (2008) | JBJS Br Mid-term clinical and sonographic outcome of arthroscopic repair of the rotator cuff. J Bone Joint Surg Br, 90 (2008), pp. 1341-1347. |  |  |  |  |  |  |  | x |  |  |  |  |  |  |  |  |  |  |  |  |  |  |  |
| Nho et al. (2009) | JSES  Prospective analysis of arthroscopic rotator cuff repair: Prognostic factors affecting clinical and ultrasound outcome. J Shoulder Elbow Surg 2009;18:13-20. |  |  |  |  |  |  |  | x |  |  |  | x |  |  |  |  |  |  | x | x |  |  |  |
| Cho et al. (2013) | JSES  Is shoulder pain for three months or longer correlated with depression, anxiety, and sleep disturbance? J Shoulder Elbow Surg 2013;22:222-228. |  |  |  |  |  |  |  |  | x |  |  |  |  |  |  |  |  |  |  |  |  |  |  |
| Potter et al. (2014) | CORR Psychological distress negatively affects self-assessment of shoulder function in patients with rotator cuff tears. Clin Orthop Relat Res, 472 (2014), pp. 3926-3932. |  |  |  |  |  |  |  |  | x |  |  |  |  |  |  |  |  |  |  |  |  |  |  |
| Ravindra et al. (2018) | JSES A prospective evaluation of predictors of pain after arthroscopic rotator cuff repair: Psychosocial factors have a stronger association than structural factors. J Shoulder Elbow Surg 2018;27:1824-1829. |  |  |  |  |  |  |  |  | x |  |  |  |  |  |  |  |  | x |  |  |  |  |  |
| Razmjou et al. (2006) | Arthroscopy Gender differences in quality of life and extent of rotator cuff pathology. Arthroscopy 2006;22:57-62. |  |  |  |  |  |  |  |  | x |  |  |  |  |  |  |  |  |  |  |  |  |  |  |
| Tashjian et al. (2007) | JSES  Factors influencing patient satisfaction after rotator cuff repair. J Shoulder Elbow Surg 2007;16:752-758. |  |  |  |  |  |  |  |  | x |  |  |  |  |  |  |  |  |  | x |  |  |  |  |
| Wylie et al. (2018) | OJSM A comprehensive evaluation of factors affecting healing, range of motion, strength, and patient-reported outcomes after arthroscopic rotator cuff repair. Orthop J Sport Med 2018;6:2325967117750104. |  |  |  |  |  |  |  |  | x |  |  |  |  |  |  |  |  |  |  |  |  |  |  |
| Castagna et al. (2008) | KSSTA Arthroscopic repair of rotator cuff tear with a modified Mason-Allen stitch: mid-term clinical and ultrasound outcomes. Knee Surg Sports Traumatol Arthrosc. 2008; 16(5): 497-503. |  |  |  |  |  |  |  |  |  | x |  |  |  |  |  |  |  |  |  |  |  |  |  |
| Demirors et al. (2010) | Int Orthop  Correlations of isokinetic measurements with tendon healing following open repair of rotator cuff tears. Int Orthop. 2010; 34(4): 531-536. |  |  |  |  |  |  |  |  |  | x |  |  |  |  |  |  |  |  | x |  |  |  |  |
| Deniz et al. (2014) | AOTS Fatty degeneration and atrophy of the rotator cuff muscles after arthroscopic repair: does it improve, halt or deteriorate? Arch Orthop Trauma Surg. 2014; 134(7): 985-990. |  |  |  |  |  |  |  |  |  | x |  |  |  |  |  |  |  |  |  |  |  |  |  |
| Gerber et al. (2009) | JSES Neer award 2007: Reversion of structural muscle changes caused by chronic rotator cuff tears using continuous musculotendinous traction. An experimental study in sheep. J Shoulder Elbow Surg. 2009; 18(2): 163-171. |  |  |  |  |  |  |  |  |  | x |  |  |  |  |  |  |  |  |  |  |  |  |  |
| Goutallier et al. (2006) | JSES Tension-free cuff repairs with excision of macroscopic tendon lesions and muscular advancement: results in a prospective series with limited fatty muscular degeneration. J Shoulder Elbow Surg. 2006; 15(2): 164-172. |  |  |  |  |  |  |  |  |  | x |  |  |  |  |  |  |  |  | x |  |  |  |  |
| Grasso et al. (2009) | Arthroscopy Single-row versus double-row arthroscopic rotator cuff repair: a prospective randomized clinical study. Arthroscopy. 2009; 25(1): 4-12. |  |  |  |  |  |  |  |  |  | x |  | x | x |  |  |  |  |  | x |  |  |  |  |
| Liem et al. (2007) | JBJS. Magnetic resonance imaging of arthroscopic supraspinatus tendon repair. J Bone Joint Surg Am. 2007; 89(8): 1770-1776. |  |  |  |  |  |  |  |  |  | x |  |  |  |  |  |  |  |  |  |  |  |  |  |
| Mellado et al. (2006) | Eur Radiol MR assessment of the repaired rotator cuff: prevalence, size, location, and clinical relevance of tendon rerupture. Eur Radiol. 2006; 16(10): 2186-2196. |  |  |  |  |  |  |  |  |  | x |  |  |  |  |  |  |  |  | x |  |  |  |  |
| Milano et al. (2010) | Arthroscopy Arthroscopic rotator cuff repair with metal and biodegradable suture anchors: a prospective randomized study. Arthroscopy. 2010; 26(9 Suppl): S112-S119. |  |  |  |  |  |  |  |  |  | x |  |  |  |  |  |  |  |  |  |  |  |  |  |
| Kew et al. (2019) | AJSM The timing of corticosteroid injections after arthroscopic shoulder procedures affects postoperative infection risk Am J Sports Med, 47 (2019), pp. 915-921. |  |  |  |  |  |  |  |  |  |  | x |  |  |  |  |  |  |  |  |  |  |  |  |
| Kim et al. (2019) | AJSM  Is it safe to inject corticosteroids into the glenohumeral joint after arthroscopic rotator cuff repair? Am J Sports Med (2019) 363546519843910 |  |  |  |  |  |  |  |  |  |  | x |  |  |  |  |  |  |  |  |  |  |  |  |
| Forsythe et al. (2019) | JBJS  The timing of injections prior to arthroscopic rotator cuff repair impacts the risk of surgical site infection J Bone Joint Surg Am, 101 (2019), pp. 682-687. |  |  |  |  |  |  |  |  |  |  | x |  |  |  |  |  |  |  |  |  |  |  |  |
| Balyk et al. (2008) | CORR  Do outcomes differ after rotator cuff repair for patients receiving workers' compensation? Clin Orthop Relat Res, 466 (2008), pp. 3025-3033. |  |  |  |  |  |  |  |  |  |  |  | x |  |  |  |  |  |  | x | x |  |  |  |
| Henn et al. (2008) | JBJS  Patients with workers' compensation claims have worse outcomes after rotator cuff repair J Bone Joint Surg Am, 90 (2008), pp. 2105-2113. |  |  |  |  |  |  |  |  |  |  |  | x |  |  |  |  |  |  | x |  |  |  |  |
| Kluger et al. (2011) | AJSM  Long-term survivorship of rotator cuff repairs using ultrasound and magnetic resonance imaging analysis Am J Sports Med, 39 (2011), pp. 2071-2081. |  |  |  |  |  |  |  |  |  |  |  | x |  |  |  |  |  |  | x |  |  |  |  |
| Namdari et al. (2010) | JSES  Does obesity affect early outcome of rotator cuff repair? J Shoulder Elbow Surg, 19 (2010), pp. 1250-1255. |  |  |  |  |  |  |  |  |  |  |  | x |  |  |  |  |  |  | x |  |  |  |  |
| Lafosse et al. (2007) | JBJS  The outcome and structural integrity of arthroscopic rotator cuff repair with use of the double-row suture anchor technique J Bone Joint Surg Am, 89 (2007), pp. 1533-1541. |  |  |  |  |  |  |  |  |  |  |  | x |  |  |  |  |  |  | x |  |  |  |  |
| Tashjian et al. (2010) | AJSM Factors affecting healing rates after arthroscopic double-row rotator cuff repair Am J Sports Med, 38 (2010), pp. 2435-2442. |  |  |  |  | x |  |  |  |  |  |  | x |  |  |  |  |  |  | x | x |  |  | x |
| Franceschi et al. (2007) | AJSM Equivalent clinical results of arthroscopic single-row and double-row suture anchor repair for rotator cuff tears: a randomized controlled trial Am J Sports Med, 35 (2007), pp. 1254-1260. |  |  |  |  |  |  |  |  |  |  |  |  | x |  |  |  |  |  |  |  |  |  |  |
| Aydin et al. (2010) | JSES  Single-row versus double-row arthroscopic rotator cuff repair in small- to medium-sized tears J Shoulder Elbow Surg, 19 (2010), pp. 722-725. |  |  |  |  |  |  |  |  |  |  |  |  | x |  |  |  |  |  |  |  |  |  |  |
| Carbonel et al. (2012) | Int Orthop  Single-row versus double-row arthroscopic repair in the treatment of rotator cuff tears: a prospective randomized clinical study Int Orthop, 36 (2012), pp. 1877-1883. |  |  |  |  |  |  |  |  |  |  |  |  | x | x |  |  |  |  |  |  |  |  |  |
| Nicholas et al. (2016) | OJSM Functional outcomes after double-row versus single-row rotator cuff repair: a prospective randomized trial Orthop J Sports Med. 2016 Oct 3;4(10):2325967116667398. |  |  |  |  |  |  |  |  |  |  |  |  | x |  |  |  |  |  |  |  |  |  |  |
| Barber (2016) | Arthroscopy  Triple-loaded single-row versus suture-bridge double-row rotator cuff tendon repair with platelet-rich plasma fibrin membrane: a randomized controlled trial Arthroscopy, 32 (2016), pp. 753-761. |  |  |  |  |  |  |  |  |  |  |  |  | x |  |  |  |  |  |  |  |  |  |  |
| Franceschi et al. (2016) | AJSM  Double-row repair lowers the retear risk after accelerated rehabilitation Am J Sports Med, 44 (2016), pp. 948-956. |  |  |  |  |  |  |  |  |  |  |  |  | x |  |  |  |  |  |  |  |  |  |  |
| Wade et al. (2017) | J Orthop Clinico-radiological evaluation of retear rate in arthroscopic double row versus single row repair technique in full thickness rotator cuff tear J Orthop, 14 (2017), pp. 313-318. |  |  |  |  |  |  |  |  |  |  |  |  | x |  |  |  |  |  |  |  |  |  |  |
| Iman et al. (2020) | JSES Three-year functional outcome of transosseous-equivalent double-row vs. single-row repair of small and large rotator cuff tears: a double-blinded randomized controlled trial J Shoulder Elbow Surg, 29 (2020), pp. 2015-2026. |  |  |  |  |  |  |  |  |  |  |  |  | x |  |  |  |  |  |  |  |  |  |  |
| Gartsman et al. (2013) | JSES Ultrasound evaluation of arthroscopic full-thickness supraspinatus rotator cuff repair: single-row versus double-row suture bridge (transosseous equivalent) fixation. Results of a prospective, randomized study. J Shoulder Elbow Surg, 22 (2013), pp. 1480-1487. |  |  |  |  |  |  |  |  |  |  |  |  | x |  |  |  |  |  |  |  |  |  |  |
| Baverel et al. (2021) | JSES Short-term outcomes of arthroscopic partial repair vs. latissimus dorsi tendon transfer in patients with massive and partially repairable rotator cuff tears. J Shoulder Elbow Surg, 30 (2021), pp. 282-289. |  |  |  |  |  |  |  |  |  |  |  |  | x |  |  |  |  |  |  |  |  |  |  |
| Ozturk et al. (2021) | JSES Prospective, randomized evaluation of latissimus dorsi transfer and superior capsular reconstruction in massive, irreparable rotator cuff tears. J Shoulder Elbow Surg, 30 (2021), pp. 1561-1571. |  |  |  |  |  |  |  |  |  |  |  |  | x |  |  |  |  |  |  |  |  |  |  |
| Paribelli et al. (2015) | Musculoskelet Surg Clinical outcome of latissimus dorsi tendon transfer and partial cuff repair in irreparable postero-superior rotator cuff tear. Musculoskelet Surg, 99 (2015), pp. 127-132. |  |  |  |  |  |  |  |  |  |  |  |  | x |  |  |  |  |  |  |  |  |  |  |
| Woodmass et al. (2020) | J ISAKOS  Arthroscopic lower trapezius tendon transfer provides equivalent outcomes to latissimus dorsi transfer in the treatment of massive posterosuperior rotator cuff tears. J ISAKOS, 5 (2020), pp. 269-274. |  |  |  |  |  |  |  |  |  |  |  |  | x |  |  |  |  |  |  |  |  |  |  |
| Duncan et al. (2015) | JSES Surgery within 6 months of an acute rotator cuff tear significantly improves outcome J Shoulder Elbow Surg, 24 (2015), pp. 1876-1880. |  |  |  |  |  |  |  |  |  |  |  |  | x |  |  |  |  |  |  |  |  |  |  |
| Hantes et al. (2011) | KSSTA  A comparison of early versus delayed repair of traumatic rotator cuff tears. Knee Surg Sports Traumatol Arthrosc, 19 (2011), pp. 1766-1770. |  |  |  |  |  |  |  |  |  |  |  |  | x |  |  |  |  |  |  |  |  |  |  |
| Petersen et al. (2011) | JSES  The timing of rotator cuff repair for the restoration of function. J Shoulder Elbow Surg, 20 (2011), pp. 62-68. |  |  |  |  |  |  |  |  |  |  |  |  | x |  |  |  |  |  |  |  |  |  |  |
| Zhaeentan et al. (2016) | KSSTA Similar results comparing early and late surgery in open repair of traumatic rotator cuff tears. Knee Surg Sports Traumatol Arthrosc, 24 (2016), pp. 3899-3906. |  |  |  |  |  |  |  |  |  |  |  |  | x |  |  |  |  |  |  |  |  |  |  |
| D'Ambrosi et al. (2016) | Musculoskelet Surg Platelet-rich plasma supplementation in arthroscopic repair of full-thickness rotator cuff tears: a randomized clinical trial. Musculoskelet Surg. 2016;100(Suppl 1):25–32. |  |  |  |  |  |  |  |  |  |  |  |  |  | x |  |  |  |  |  |  |  |  |  |
| Kim et al. (2016) | AJSM Conventional En masse repair versus separate double-layer double-row repair for the treatment of delaminated rotator Cuff tears. Am J Sports Med. 2016;44(5):1146–1152. |  |  |  |  |  |  |  |  |  |  |  |  |  | x |  |  |  |  |  |  |  |  |  |
| Malavolta et al. (2018) | AJSM Clinical and structural evaluations of rotator Cuff repair with and without added platelet-rich plasma at 5-year follow-up: a prospective randomized study. Am J Sports Med. 2018;46(13):3134–3141. |  |  |  |  |  |  |  |  |  |  |  |  |  | x |  |  |  |  |  |  |  |  |  |
| Pandey et al. (2016) | JSES Does application of moderately concentrated platelet-rich plasma improve clinical and structural outcome after arthroscopic repair of medium-sized to large rotator cuff tear? A randomized controlled trial. J Shoulder Elb Surg. 2016;25(8):1312–1322. |  |  |  |  |  |  |  |  |  |  |  |  |  | x |  |  |  |  |  |  |  |  |  |
| Randelli et al. (2017) | AJSM Advantages of arthroscopic rotator Cuff repair with a Transosseous suture technique: a prospective randomized controlled trial. Am J Sports Med. 2017;45(9):2000–2009. |  |  |  |  |  |  |  |  |  |  |  |  |  | x |  |  |  |  |  |  |  |  |  |
| Barber et al. (2012) | Arthroscopy  A prospective, randomized evaluation of acellular human dermal matrix augmentation for arthroscopic rotator cuff repair. Arthroscopy. 2012;28(1):8–15. |  |  |  |  |  |  |  |  |  |  |  |  |  | x |  |  |  |  |  |  |  |  |  |
| Cai et al. (2018) | AJSM Arthroscopic rotator Cuff repair with graft augmentation of 3-dimensional biological collagen for moderate to large tears: a randomized controlled study. Am J Sports Med. 2018;46(6):1424–1431. |  |  |  |  |  |  |  |  |  |  |  |  |  | x |  |  |  |  |  |  |  |  |  |
| Avanzi et al. (2019) | JSES Prospective randomized controlled trial for patch augmentation in rotator cuff repair: 24-month outcomes. J Shoulder Elb Surg. 2019;28(10):1918–1927. |  |  |  |  |  |  |  |  |  |  |  |  |  | x |  |  |  |  |  |  |  |  |  |
| Iannotti et al. (2006) | JBJS  Porcine small intestine submucosa augmentation of surgical repair of chronic two-tendon rotator cuff tears. A randomized, controlled trial. J Bone Joint Surg Am. 2006;88(6):1238–1244. |  |  |  |  |  |  |  |  |  |  |  |  |  | x |  |  |  |  |  |  |  |  |  |
| Koh et al. (2014) | JBJS Effect of immobilization without passive exercise after rotator cuff repair: randomized clinical trial comparing four and eight weeks of immobilization. J Bone Joint Surg Am. 2014;96(6):e44. |  |  |  |  |  |  |  |  |  |  |  |  |  | x |  |  |  |  |  |  |  |  |  |
| Sheps et al. (2019) | Arthroscopy  Early Active Motion Versus Sling Immobilization After Arthroscopic Rotator Cuff Repair: A Randomized Controlled Trial. Arthroscopy. 2019;35(3):749–760.e742. |  |  |  |  |  |  |  |  |  |  |  |  |  | x |  |  |  |  |  |  |  |  |  |
| Lee et al. (2016) | JSES Surgical treatment of lesions of the long head of the biceps brachii tendon with rotator cuff tear: a prospective randomized clinical trial comparing the clinical results of tenotomy and tenodesis. J Shoulder Elb Surg. 2016;25(7):1107–1114. |  |  |  |  |  |  |  |  |  |  |  |  |  | x |  |  |  |  |  |  |  |  |  |
| Nam et al. (2018) | Arthroscopy  Outcomes after limited or extensive Bursectomy during rotator Cuff repair: randomized controlled trial. Arthroscopy. 2018;34(12):3167–3174. |  |  |  |  |  |  |  |  |  |  |  |  |  | x |  |  |  |  |  |  |  |  |  |
| Osti et al. (2013) | Int Orthop Microfractures at the rotator cuff footprint: a randomised controlled study. Int Orthop. 2013;37(11):2165–2171. |  |  |  |  |  |  |  |  |  |  |  |  |  | x |  |  |  |  |  |  |  |  |  |
| Liu et al. (2017) | Medicine Comparison of clinical outcomes in all-arthroscopic versus mini-open repair of rotator cuff tears: a randomized clinical trial. Medicine (Baltimore) 2017;96(11):e6322. |  |  |  |  |  |  |  |  |  |  |  |  |  | x |  |  |  |  |  |  |  |  |  |
| Rhee et al. (2012) | AJSM Arthroscopic rotator cuff repair using modified Mason-Allen medial row stitch: knotless versus knot-tying suture bridge technique. Am J Sports Med. 2012;40(11):2440–2447. |  |  |  |  |  |  |  |  |  |  |  |  |  | x |  |  |  |  |  |  |  |  |  |
| Miyatake et al. (2018) | KSSTA  Comparable clinical and structural outcomes after arthroscopic rotator cuff repair in diabetic and non-diabetic patients. Knee Surg. Sports Traumatol. Arthrosc., 2018, 26(12), 3810-3817. |  |  |  |  |  |  |  |  |  |  |  |  |  |  | x |  |  |  |  |  |  |  |  |
| Chen et al. (2003) | JSES  Rotator cuff repair in patients with type I diabetes mellitus. J.Shoulder Elbow Surg., 2003, 12(5), 416-421. |  |  |  |  |  |  |  |  |  |  |  |  |  |  | x |  |  |  |  |  |  |  |  |
| Dhar et al. (2013) | Phys. Sportsmed.  Arthroscopic rotator cuff repair: impact of diabetes mellitus on patient outcomes. Phys. Sportsmed., 2013, 41(1), 22-29. |  |  |  |  |  |  |  |  |  |  |  |  |  |  | x |  |  |  |  |  |  |  |  |
| Kim et al. (2018) | JSES Factors affecting rotator cuff integrity after arthroscopic repair for medium-sized or larger cuff tears: a retrospective cohort study. J. Shoulder Elbow Surg., 2018, 27(6), 1012-1020. |  |  |  |  |  |  |  |  |  |  |  |  |  |  | x |  |  |  |  |  |  |  | x |
| Clement et al. (2010) | JBJS Does diabetes affect outcome after arthroscopic repair of the rotator cuff? J. Bone Joint Surg. Br., 2010, 92(8), 1112-1117. |  |  |  |  |  |  |  |  |  |  |  |  |  |  | x |  |  |  |  |  |  |  |  |
| Hsu et al. (2007) | J Formos Med Assoc Surgical results in rotator cuff tears with shoulder stiffness. J. Formos. Med. Assoc., 2007, 106(6), 452-461. |  |  |  |  |  |  |  |  |  |  |  |  |  |  | x |  |  |  |  |  |  |  |  |
| Cho et al. (2015) | AJSM  The influence of diabetes mellitus on clinical and structural outcomes after arthroscopic rotator cuff repair. Am. J. Sports Med., 2015, 43(4), 991-997. |  |  |  |  |  |  |  |  |  |  |  |  |  |  | x |  |  |  |  |  |  |  |  |
| Assunção et al. (2017) | Clin Orthop Relat Res Matrix metalloproteases 1 and 3 promoter gene polymorphism is associated with rotator cuff tear Clin Orthop Relat Res, 475 (2017), pp. 1904-1910. |  |  |  |  |  |  |  |  |  |  |  |  |  |  |  |  | x |  |  |  |  |  |  |
| Bonato et al. (2016) | Int J Oral Maxillofac Surg ESRRB polymorphisms are associated with comorbidity of temporomandibular disorders and rotator cuff disease. Int J Oral Maxillofac Surg, 45 (2016), pp. 323-331. |  |  |  |  |  |  |  |  |  |  |  |  |  |  |  |  | x |  |  |  |  |  |  |
| Kluger et al. (2017) | J Orthop Res  Candidate gene approach identifies six SNPs in tenascin-C (TNC) associated with degenerative rotator cuff tears. J Orthop Res, 35 (2017), pp. 894-901. |  |  |  |  |  |  |  |  |  |  |  |  |  |  |  |  | x |  |  |  |  |  |  |
| Longo et al. (2018) | BMC Med Genet Genetics of rotator cuff tears: no association of col5a1 gene in a case-control study. BMC Med Genet, 19 (2018), p. 217. |  |  |  |  |  |  |  |  |  |  |  |  |  |  |  |  | x |  |  |  |  |  |  |
| Motta et al. (2014) | JSES Evidence of genetic variations associated with rotator cuff disease. J Shoulder Elbow Surg, 23 (2014), pp. 227-235. |  |  |  |  |  |  |  |  |  |  |  |  |  |  |  |  | x |  |  |  |  |  |  |
| Peach et al. (2007) | Clin Orthop Relat Res Cuff tear arthropathy: evidence of functional variation in pyrophosphate metabolism genes. Clin Orthop Relat Res, 462 (2007), pp. 67-72. |  |  |  |  |  |  |  |  |  |  |  |  |  |  |  |  | x |  |  |  |  |  |  |
| Tashjian et al. (2016) | JSES Genome-wide association study for rotator cuff tears identifies two significant single-nucleotide polymorphisms. J Shoulder Elbow Surg, 25 (2016), pp. 174-179. |  |  |  |  |  |  |  |  |  |  |  |  |  |  |  |  | x |  |  |  |  |  |  |
| Teerlink et al. (2015) | JSES Significant association of full-thickness rotator cuff tears and estrogen-related receptor-beta (ESRRB). J Shoulder Elbow Surg, 24 (2015), pp. e31-e35. |  |  |  |  |  |  |  |  |  |  |  |  |  |  |  |  | x |  |  |  |  |  |  |
| Abrams et al. (2016) | JSES Association of synovial inflammation and inflammatory mediators with glenohumeral rotator cuff pathology. J Shoulder Elbow Surg, 25 (2016), pp. 989-997. |  |  |  |  |  |  |  |  |  |  |  |  |  |  |  |  | x |  |  |  |  |  |  |
| Akbar et al. (2017) | RMD Open Targeting danger in human tendinopathy: the HMGB1/TLR4 axis. RMD Open, 3 (2017), p. e000456. |  |  |  |  |  |  |  |  |  |  |  |  |  |  |  |  | x |  |  |  |  |  |  |
| Belangero et al. (2018) | J Orthop Res Changes in the expression of matrix extracellular genes and TGFB family members in rotator cuff tears. J Orthop Res, 36 (2018), pp. 2542-2553. |  |  |  |  |  |  |  |  |  |  |  |  |  |  |  |  | x |  |  |  |  |  |  |
| Campbell et al. (2014) | Mediators Inflam IL-21 receptor expression in human tendinopathy. Mediators Inflam, 2014 (2014), p. 481206. |  |  |  |  |  |  |  |  |  |  |  |  |  |  |  |  | x |  |  |  |  |  |  |
| Chaudhury et al. (2016) | JSES Gene expression profiles of changes underlying different-sized human rotator cuff tendon tears. J Shoulder Elbow Surg, 25 (2016), pp. 1561-1570. |  |  |  |  |  |  |  |  |  |  |  |  |  |  |  |  | x |  |  |  |  |  |  |
| Kurzdiel et al. (2015) | J Orthop Res  The impact of rotator cuff deficiency on structure, mechanical properties, and gene expression profiles of the long head of the biceps tendon (LHBT): implications for management of the LHBT during primary shoulder arthroplasty. J Orthop Res, 33 (2015), pp. 1158-1164. |  |  |  |  |  |  |  |  |  |  |  |  |  |  |  |  | x |  |  |  |  |  |  |
| Leal et al. (2017) | PloS One  Epigenetic regulation of metalloproteinases and their inhibitors in rotator cuff tears. PloS One, 12 (2017), p. e0184141. |  |  |  |  |  |  |  |  |  |  |  |  |  |  |  |  | x |  |  |  |  |  |  |
| Lundgreen et al. (2011) | Br J Sports Med Tenocyte apoptosis in the torn rotator cuff: a primary or secondary pathological event?. Br J Sports Med, 45 (2011), pp. 1035-1039. |  |  |  |  |  |  |  |  |  |  |  |  |  |  |  |  | x |  |  |  |  |  |  |
| Millar et al. (2008) | Clin Orthop Relat Res Heat shock protein and apoptosis in supraspinatus tendinopathy. Clin Orthop Relat Res, 466 (2008), pp. 1569-1576. |  |  |  |  |  |  |  |  |  |  |  |  |  |  |  |  | x |  |  |  |  |  |  |
| Millar et al. (2016) | Sci Rep  IL-17A mediates inflammatory and tissue remodelling events in early human tendinopathy. Sci Rep, 6 (2016), p. 27149. |  |  |  |  |  |  |  |  |  |  |  |  |  |  |  |  | x |  |  |  |  |  |  |
| Neuwirth et al. (2006) | Arthritis Res Ther Expression of bioactive bone morphogenetic proteins in the subacromial bursa of patients with chronic degeneration of the rotator cuff. Arthritis Res Ther, 8 (2006), p. R92. |  |  |  |  |  |  |  |  |  |  |  |  |  |  |  |  | x |  |  |  |  |  |  |
| Plachel et al. (2019) | J Orthop Res Risk factors for rotator cuff disease: an experimental study on intact human subscapularis tendons. J Orthop Res, 38 (2019), pp. 182-191. |  |  |  |  |  |  |  |  |  |  |  |  |  |  |  |  | x |  |  |  |  |  |  |
| Thakkar et al. (2014) | Connext Tissue Res Distribution and expression of type VI collagen and elastic fibers in human rotator cuff tendon tears. Connect Tissue Res, 55 (2014), pp. 397-402. |  |  |  |  |  |  |  |  |  |  |  |  |  |  |  |  | x |  |  |  |  |  |  |
| Ahn et al. (2017) | AJSM Differences of RNA expression in the tendon according to anatomic outcomes in rotator cuff repair. Am J Sports Med, 45 (2017), pp. 2995-3003. |  |  |  |  |  |  |  |  |  |  |  |  |  |  |  |  | x |  |  |  |  |  |  |
| Gotoh et al. (2013) | KSSTA  Increased matrix metalloprotease-3 gene expression in ruptured rotator cuff tendons is associated with postoperative tendon retear. Knee Surg Sports Traumatol Arthrosc, 21 (2013), pp. 1807-1812. |  |  |  |  |  |  |  |  |  |  |  |  |  |  |  |  | x |  |  |  |  |  |  |
| Kluger et al. (2017) | AJSM Novel tenascin-C haplotype modifies the risk for a failure to heal after rotator cuff repair. Am J Sports Med, 45 (2017), pp. 2955-2964. |  |  |  |  |  |  |  |  |  |  |  |  |  |  |  |  | x |  |  |  |  |  |  |
| Robertson et al. (2012) | AJSM Failed healing of rotator cuff repair correlates with altered collagenase and gelatinase in supraspinatus and subscapularis tendons. Am J Sports Med, 40 (2012), pp. 1993-2001. |  |  |  |  |  |  |  |  |  |  |  |  |  |  |  |  | x |  |  |  |  |  |  |
| Tashjian et al. (2016) | JSES Identification of a genetic variant associated with rotator cuff repair healing. J Shoulder Elbow Surg, 25 (2016), pp. 865-872. |  |  |  |  |  |  |  |  |  |  |  |  |  |  |  |  | x |  |  |  |  |  |  |
| Gibson et al. (2019) | JSES Intersection of catastrophizing, gender, and disease severity in preoperative rotator cuff surgical patients: A cross-sectional study. Journal of Shoulder and Elbow Surgery, 28(12), 2284–2289. |  |  |  |  |  |  |  |  |  |  |  |  |  |  |  |  |  | x |  |  |  |  |  |
| Lau et al. (2019) | AJSM The effect of preexisting and shoulder-specific depression and anxiety on patient-reported outcomes after arthroscopic rotator cuff repair. American Journal of Sports Medicine, 47(13), 3073–3079. |  |  |  |  |  |  |  |  |  |  |  |  |  |  |  |  |  | x |  |  |  |  |  |
| Sahoo et al. (2020) | AJSM Associations of preoperative patient mental health and sociodemographic and clinical characteristics with baseline pain, function, and satisfaction in patients undergoing rotator cuff repairs. American Journal of Sports Medicine, 48(2), 432–443. |  |  |  |  |  |  |  |  |  |  |  |  |  |  |  |  |  | x |  |  |  |  |  |
| Tashjian et al. (2004) | JBJS Am  The effect of comorbidity on self-assessed function in patients with a chronic rotator cuff tear. Journal of Bone and Joint Surgery-American Volume, 86(2), 355–362. |  |  |  |  |  |  |  |  |  |  |  |  |  |  |  |  |  | x |  |  |  |  |  |
| Thorpe et al. (2018) | CORR Are psychologic factors associated with shoulder scores after rotator cuff surgery? Clinical Orthopaedics and Related Research, 476(10), 2062–2073. |  |  |  |  |  |  |  |  |  |  |  |  |  |  |  |  |  | x |  |  |  |  |  |
| Ames et al. (2012) | JBJS Association between acromial index and outcomes following arthroscopic repair of full-thickness rotator cuff tears. J Bone Joint Surg Am 94:1862–1869. |  |  |  |  |  |  |  |  |  |  |  |  |  |  |  |  |  |  | x |  |  |  |  |
| Bishop et al. (2006) | JSES Cuff integrity after arthroscopic versus open rotator cuff repair: a prospective study. J Shoulder Elbow Surg 15:290–299. |  |  |  |  |  |  |  |  |  |  |  |  |  |  |  |  |  |  | x |  |  |  |  |
| Boissonnault et al. (2007) | J Orthop Sports Phys Ther Patient outcome following rehabilitation for rotator cuff repair surgery: the impact of selected medical comorbidities. J Orthop Sports Phys Ther 37:312–319. |  |  |  |  |  |  |  |  |  |  |  |  |  |  |  |  |  |  | x |  |  |  |  |
| Charousset et al. (2008) | Arthroscopy The time for functional recovery after arthroscopic rotator cuff repair: correlation with tendon healing controlled by computed tomography arthrography. Arthroscopy 24:25–33. |  |  |  |  |  |  |  |  |  |  |  |  |  |  |  |  |  |  | x |  |  |  |  |
| Di Paola (2013) | JSES Limited physical therapy utilization protocol does not affect impairment and disability in Workers’ Compensation patients after rotator cuff repair: a short-term follow-up study. J Shoulder Elbow Surg 22:409–417. |  |  |  |  |  |  |  |  |  |  |  |  |  |  |  |  |  |  | x |  |  |  |  |
| Ellman et al. (1986) | JBJS Repair of the rotator cuff. End-result study of factors influencing reconstruction. J Bone Joint Surg Am 68:1136–1144. |  |  |  |  |  |  |  |  |  |  |  |  |  |  |  |  |  |  | x |  |  |  |  |
| Flurin et al. (2013) | Orthop Traumatol Surg Res Arthroscopic repair of the rotator cuff: prospective study of tendon healing after 70 years of age in 145 patients. Orthop Traumatol Surg Res 99:S379–S384. |  |  |  |  |  |  |  |  |  |  |  |  |  |  |  |  |  |  | x |  |  |  |  |
| Flurin et al. (2007) | Arthrosc J Arthrosc Relat Surg Cuff integrity after arthroscopic rotator cuff repair: correlation with clinical results in 576 cases. Arthrosc J Arthrosc Relat Surg 23:340–346. |  |  |  |  |  |  |  |  |  |  |  |  |  |  |  |  |  |  | x |  |  |  |  |
| Goutallier et al. (2003) | JSES Influence of cuff muscle fatty degeneration on anatomic and functional outcomes after simple suture of full-thickness tears. J Shoulder Elbow Surg 12:550–554. |  |  |  |  |  |  |  |  |  |  |  |  |  |  |  |  |  |  | x |  |  |  |  |
| Iannotti et al. (1996) | JSES Postoperative assessment of shoulder function: a prospective study of full-thickness rotator cuff tears. J Shoulder Elbow Surg 5:449–457. |  |  |  |  |  |  |  |  |  |  |  |  |  |  |  |  |  |  | x |  |  |  |  |
| Jo and Shin (2013) | JBJS Cross-sectional area of the supraspinatus muscle after rotator cuff repair: an anatomic measure of outcome. J Bone Joint Surg Am 95:1785–1791. |  |  |  |  |  |  |  |  |  |  |  |  |  |  |  |  |  |  | x |  |  |  |  |
| Kim et al. (2013) | AJSM Treatment outcomes of single- versus double-row repair for larger than medium-sized rotator cuff tears: the effect of preoperative remnant tendon length. Am J Sports Med 41:2270–2277. |  |  |  |  |  |  |  |  |  |  |  |  |  |  |  |  |  |  | x |  |  |  |  |
| Koh et al. (2014) | AJSM Preoperative factors affecting footprint coverage in rotator cuff repair. Am J Sports Med 42:869–876. |  |  |  |  |  |  |  |  |  |  |  |  |  |  |  |  |  |  | x |  |  |  |  |
| Lam and Mok (2004) | JSES Open repair of massive rotator cuff tears in patients aged sixty-five years or over: is it worthwhile? J Shoulder Elbow Surg 13:517–521. |  |  |  |  |  |  |  |  |  |  |  |  |  |  |  |  |  |  | x |  |  |  |  |
| Lee et al. (2013) | CORR Clinical and radiological evaluation after arthroscopic rotator cuff repair using suture bridge technique. Clin Orthop Relat Res Surg 5:306–313. |  |  |  |  |  |  |  |  |  |  |  |  |  |  |  |  |  |  | x |  |  |  |  |
| Lichtenberg et al. (2006) | KSSTA Influence of tendon healing after arthroscopic rotator cuff repair on clinical outcome using single-row Mason-Allen suture technique: a prospective, MRI controlled study. Knee Surg Sports Traumatol Arthrosc 14:1200–1206. |  |  |  |  |  |  |  |  |  |  |  |  |  |  |  |  |  |  | x |  |  |  |  |
| Melean et al. (2013) | Int Orthop The acromial index is not predictive for failed rotator cuff repair. Int Orthop 37:2173–2179. |  |  |  |  |  |  |  |  |  |  |  |  |  |  |  |  |  |  | x |  |  |  |  |
| Mellado et al. (2005) | AJR Am J Roentgenol Surgically repaired massive rotator cuff tears: MRI of tendon integrity, muscle fatty degeneration, and muscle atrophy correlated with intraoperative and clinical findings. AJR Am J Roentgenol 184:1456–1463. |  |  |  |  |  |  |  |  |  |  |  |  |  |  |  |  |  |  | x |  |  |  |  |
| Meyer et al. (2012) | AJSM Retraction of supraspinatus muscle and tendon as predictors of success of rotator cuff repair. Am J Sports Med 40:2242–2247. |  |  |  |  |  |  |  |  |  |  |  |  |  |  |  |  |  |  | x |  |  |  |  |
| Milano et al. (2013) | Arthroscopy Efficacy of marrow-stimulating technique in arthroscopic rotator cuff repair: a prospective randomized study. Arthroscopy 29:802–810. |  |  |  |  |  |  |  |  |  |  |  |  |  |  |  |  |  |  | x |  |  |  |  |
| Nho et al. (2009) | JSES Prospective analysis of arthroscopic rotator cuff repair: subgroup analysis. J Shoulder Elbow Surg 18:697–704. |  |  |  |  |  |  |  |  |  |  |  |  |  |  |  |  |  |  | x |  |  |  |  |
| Oh et al. (2010) | AJSM Effect of age on functional and structural outcome after rotator cuff repair. Am J Sports Med 38:672–678. |  |  |  |  |  |  |  |  |  |  |  |  |  |  |  |  |  |  | x |  |  |  |  |
| Ozbaydar et al. (2007) | Acta Orthop Traumatol Turc Athroscopic rotator cuff repair: evaluation of outcomes and analysis of prognostic factors. Acta Orthop Traumatol Turc 41:169–174. |  |  |  |  |  |  |  |  |  |  |  |  |  |  |  |  |  |  | x |  |  |  |  |
| Pai and Lawson (2001) | JSES Rotator cuff repair in a district hospital setting: outcomes and analysis of prognostic factors. J Shoulder Elbow Surg 10:236–241. |  |  |  |  |  |  |  |  |  |  |  |  |  |  |  |  |  |  | x |  |  |  |  |
| Park et al. (2013) | Arthroscopy Clinical and ultrasonographic outcomes of arthroscopic suture bridge repair for massive rotator cuff tear. Arthroscopy 29:280–289. |  |  |  |  |  |  |  |  |  |  |  |  |  |  |  |  |  |  | x |  |  |  |  |
| Park et al. (2010) | CORR Does an arthroscopic suture bridge technique maintain repair integrity? A serial evaluation by ultrasonography. Clin Orthop Relat Res 468:1578–1587. |  |  |  |  |  |  |  |  |  |  |  |  |  |  |  |  |  |  | x |  |  |  |  |
| Prasad et al. (2005) | Acta Orthop Belg Outcome of open rotator cuff repair. An analysis of risk factors. Acta Orthop Belg 71:662–666. |  |  |  |  |  |  |  |  |  |  |  |  |  |  |  |  |  |  | x | x |  |  |  |
| Robinson et al. (2013) | Bone Joint J  Rotator cuff repair in patients over 70 years of age: early outcomes and risk factors associated with re-tear. Bone Joint J 95-B:199–205. |  |  |  |  |  |  |  |  |  |  |  |  |  |  |  |  |  |  | x |  |  |  |  |
| Romeo et al. (1999) | CORR Repair of full thickness rotator cuff tears. Gender, age, and other factors affecting outcome. Clin Orthop Relat Res 367:243–255. |  |  |  |  |  |  |  |  |  |  |  |  |  |  |  |  |  |  | x |  |  |  |  |
| Sherman et al. (2008) | CORR Risk factors for readmission and revision surgery following rotator cuff repair. Clin Orthop Relat Res 466:608–613. |  |  |  |  |  |  |  |  |  |  |  |  |  |  |  |  |  |  | x |  |  |  |  |
| Tashjian et al. (2013) | Arthroscopy Influence of preoperative musculotendinous junction position on rotator cuff healing using single-row technique. Arthroscopy 29:1748–1754. |  |  |  |  |  |  |  |  |  |  |  |  |  |  |  |  |  |  | x |  |  |  |  |
| Vad et al. (2002) | Clin J Sport Med Negative prognostic factors in managing massive rotator cuff tears. Clin J Sport Med 12:151–157. |  |  |  |  |  |  |  |  |  |  |  |  |  |  |  |  |  |  | x |  |  |  |  |
| Van Linthoudt et al. (2003) | Joint Bone Spine Rotator cuff repair. Long-term results. Joint Bone Spine 70:271–275. |  |  |  |  |  |  |  |  |  |  |  |  |  |  |  |  |  |  | x |  |  |  |  |
| Voigt et al. (2010) | AJSM Arthroscopic supraspinatus tendon repair with suture-bridging technique: functional outcome and magnetic resonance imaging. Am J Sports Med 38:983–991. |  |  |  |  |  |  |  |  |  |  |  |  |  |  |  |  |  |  | x |  |  |  |  |
| Worland et al. (1999) | JSES Repair of massive rotator cuff tears in patients older than 70 years. J Shoulder Elbow Surg 8:26–30. |  |  |  |  |  |  |  |  |  |  |  |  |  |  |  |  |  |  | x |  |  |  |  |
| Wu et al. (2012) | AJSM Intraoperative determinants of rotator cuff repair integrity: an analysis of 500 consecutive repairs. Am J Sports Med 40:2771–2776. |  |  |  |  |  |  |  |  |  |  |  |  |  |  |  |  |  |  | x |  |  |  |  |
| Oh et al. (2009) | Arthroscopy Prognostic factors affecting anatomic outcome of rotator cuff repair and correlation with functional outcome. Arthroscopy 25:30–39. |  |  |  |  |  |  |  |  |  |  |  |  |  |  |  |  |  |  | x |  |  |  |  |
| Galatz et al. (2006) | JBJS Nicotine delays tendon-to-bone healing in a rat shoulder model. J Bone Joint Surg Am. 2006;88(9):2027-2034. |  |  |  |  |  |  |  |  |  |  |  |  |  |  |  |  |  |  |  | x |  |  |  |
| Park et al. (2011) | Arthroscopy  Poly-L/D-lactic acid anchors are associated with reoperation and failure of SLAP repairs. Arthroscopy. 2011;27(10):1335-1340. |  |  |  |  |  |  |  |  |  |  |  |  |  |  |  |  |  |  |  | x |  |  |  |
| Provencher et al. (2013) | AJSM A prospective analysis of 179 type 2 superior labrum anterior and posterior repairs: outcomes and factors associated with success and failure. Am J Sports Med. 2013;41(4):880-886. |  |  |  |  |  |  |  |  |  |  |  |  |  |  |  |  |  |  |  | x |  |  |  |
| Gerber et al. (2018) | Arthroscopy  Arthroscopic correction of the critical shoulder angle through lateral acromioplasty: A safe adjunct to rotator cuff repair Arthroscopy, 34 (2018), pp. 771-780. |  |  |  |  |  |  |  |  |  |  |  |  |  |  |  |  |  |  |  |  | x |  |  |
| Gulotta et al. (2011) | JSES Prospective evaluation of arthroscopic rotator cuff repairs at 5 years: part I–functional outcomes and radiographic healing rates. J Shoulder Elbow Surg 20(6):934–940. |  |  |  |  |  |  |  |  |  |  |  |  |  |  |  |  |  |  |  |  |  | x |  |
| Marrero et al. (2011) | Arthroscopy Long-term follow-up of arthroscopic rotator cuff repair. Arthroscopy 27(7):885–888. |  |  |  |  |  |  |  |  |  |  |  |  |  |  |  |  |  |  |  |  |  | x |  |
| Denard et al. (2012) | Arthroscopy Long-term outcome of arthroscopic massive rotator cuff repair: the importance of double-row fixation. Arthroscopy 28(7):909–915. |  |  |  |  |  |  |  |  |  |  |  |  |  |  |  |  |  |  |  |  |  | x |  |
| Denard et al. (2012) | Arthroscopy Long-term outcome of a consecutive series of subscapularis tendon tears repaired arthroscopically. Arthroscopy 28(11):1587–1591. |  |  |  |  |  |  |  |  |  |  |  |  |  |  |  |  |  |  |  |  |  | x |  |
| Porcellini et al. (2011) | JSES Partial repair of irreparable supraspinatus tendon tears: clinical and radiographic evaluations at long-term follow-up. J Shoulder Elbow Surg 20(7):1170–1177. |  |  |  |  |  |  |  |  |  |  |  |  |  |  |  |  |  |  |  |  |  | x |  |
| Stuart et al. (2013) | Arthroscopy Long-term outcome for arthroscopic repair of partial articular-sided supraspinatus tendon avulsion. Arthroscopy 29(5):818–823. |  |  |  |  |  |  |  |  |  |  |  |  |  |  |  |  |  |  |  |  |  | x |  |
| Budoff et al. (2005) | Arthroscopy  Arthroscopic rotator cuff debridement without decompression for the treatment of tendinosis. Arthroscopy 21(9):1081–1089. |  |  |  |  |  |  |  |  |  |  |  |  |  |  |  |  |  |  |  |  |  | x |  |
| Kartus et al. (2006) | Arthroscopy Long-term clinical and ultrasound evaluation after arthroscopic acromioplasty in patients with partial rotator cuff tears. Arthroscopy 22(1):44–49. |  |  |  |  |  |  |  |  |  |  |  |  |  |  |  |  |  |  |  |  |  | x |  |
| Paxton et al. (2013) | JBJS Am The effect of comorbidity on self-assessed function in patients with a chronic rotator cuff tear. Journal of Bone and Joint Surgery-American Volume, 86(2), 355–362. |  |  |  |  |  |  |  |  |  |  |  |  |  |  |  |  |  |  |  |  |  | x |  |
| Le et al. (2014) | AJSM Factors predicting rotator cuff retears: an analysis of 1000 consecutive rotator cuff repairs Am J Sports Med, 42 (2014), pp. 1134-1142. |  |  |  |  |  |  |  |  |  |  |  |  |  |  |  |  |  |  |  |  |  |  | x |
| Diebold et al. (2017) | JBJS Am Relationship between age and rotator cuff retear J Bone Joint Surg Am, 99 (2017), pp. 1198-1205. |  |  |  |  |  |  |  |  |  |  |  |  |  |  |  |  |  |  |  |  |  |  | x |
| Garcia et al. (2017) | JSES Hyperlipidemia increases the risk of retear after arthroscopic rotator cuff repair J Shoulder Elbow Surg, 26 (2017), pp. 2086-2090. |  |  |  |  |  |  |  |  |  |  |  |  |  |  |  |  |  |  |  |  |  |  | x |
| Jeong et al. (2018) | AJSM Factors predictive of healing in large rotator cuff tears: is it possible to predict retear preoperatively? Am J Sports Med, 46 (2018), pp. 1693-1700. |  |  |  |  |  |  |  |  |  |  |  |  |  |  |  |  |  |  |  |  |  |  | x |
| Kim and Kim (2016) | Arthroscopy Risk factors for retear after arthroscopic repair of full-thickness rotator cuff tears using the suture bridge technique: classification system Arthroscopy, 32 (2016), pp. 2191-2200. |  |  |  |  |  |  |  |  |  |  |  |  |  |  |  |  |  |  |  |  |  |  | x |
| Park et al. (2015) | AJSM Prognostic factors affecting rotator cuff healing after arthroscopic repair in small to medium-sized tears Am J Sports Med, 43 (2015), pp. 2386-2392. |  |  |  |  |  |  |  |  |  |  |  |  |  |  |  |  |  |  |  |  |  |  | x |
| Kwon et al. (2018) | AJSM The rotator cuff healing index: a new scoring system to predict rotator cuff healing after surgical repair Am J Sports Med, 47 (2018), pp. 173-180. |  |  |  |  |  |  |  |  |  |  |  |  |  |  |  |  |  |  |  |  |  |  | x |
| Harada et al. (2021) | JSES Combination of risk factors affecting retear after arthroscopic rotator cuff repair: a decision tree analysis J Shoulder Elbow Surg, 30 (2021), pp. 9-15. |  |  |  |  |  |  |  |  |  |  |  |  |  |  |  |  |  |  |  |  |  |  | x |
| Namdari et al. (2014) | JBJS Factors affecting outcome after structural failure of repaired rotator cuff tears J Bone Joint Surg Am, 96 (2014), pp. 99-105. |  |  |  |  |  |  |  |  |  |  |  |  |  |  |  |  |  |  |  |  |  |  | x |
| Lee et al. (2017) | AJSM Evaluation of the risk factors for a rotator cuff retear after repair surgery Am J Sports Med, 45 (2017), pp. 1755-1761. |  |  |  |  |  |  |  |  |  |  |  |  |  |  |  |  |  |  |  |  |  |  | x |
